# Small-scale phenotypic differentiation along complex stream gradients in a non-native amphipod

**DOI:** 10.1186/s12983-019-0327-8

**Published:** 2019-07-11

**Authors:** Jonas Jourdan, Kathrin Piro, Alexander Weigand, Martin Plath

**Affiliations:** 1 Department of Aquatic Ecotoxicology, Institute for Ecology, Evolution and Diversity, Goethe University Frankfurt am Main, Frankfurt am Main, Germany; 2 Department of River Ecology and Conservation, Senckenberg Research Institute and Natural History Museum Frankfurt, Gelnhausen, Germany; 3 National Museum of Natural History Luxembourg, Luxembourg City, Luxembourg; 4 College of Animal Science and Technology, Northwest A&F University, Yangling, People’s Republic of China; 5 Shaanxi Key Laboratory for Molecular Biology for Agriculture, Northwest A&F University, Yangling, People’s Republic of China

**Keywords:** Local adaptation, Rapid evolution, Life-history evolution, Thermal pollution, Global warming, Intraspecific divergence, *Gammarus roeselii*, Aquatic invertebrates, Invasive species

## Abstract

**Background:**

Selective landscapes in rivers are made up by an array of selective forces that vary from source to downstream regions or between seasons, and local/temporal variation in fitness maxima can result in gradual spatio-temporal variation of phenotypic traits. This study aimed at establishing freshwater amphipods as future model organisms to study adaptive phenotypic diversification (evolutionary divergence and/or adaptive plasticity) along stream gradients.

**Methods:**

We collected *Gammarus roeselii* from 16 sampling sites in the Rhine catchment during two consecutive seasons (summer and winter). Altogether, we dissected *n* = 1648 individuals and quantified key parameters related to morphological and life-history diversification, including naturally selected (e.g., gill surface areas) as well as primarily sexually selected traits (e.g., male antennae). Acknowledging the complexity of selective regimes in streams and the interrelated nature of selection factors, we assessed several abiotic (e.g., temperature, flow velocity) and biotic ecological parameters (e.g., conspecific densities, sex ratios) and condensed them into four principal components (PCs).

**Results:**

Generalized least squares models revealed pronounced phenotypic differentiation in most of the traits investigated herein, and components of the stream gradient (PCs) explained parts of the observed differences. Depending on the trait under investigation, phenotypic differentiation could be ascribed to variation in abiotic conditions, anthropogenic disturbance (influx of thermally polluted water), or population parameters. For example, female fecundity showed altitudinal variation and decreased with increasing conspecific densities, while sexual dimorphism in the length of male antennae—used for mate finding and assessment—increased with increasing population densities and towards female-biased sex ratios.

**Conclusions:**

We provide a comprehensive protocol for comparative analyses of intraspecific variation in life history traits in amphipods. Whether the observed phenotypic differentiation over small geographical distances reflects evolutionary divergence or plasticity (or both) remains to be investigated in future studies. Independent of the mechanisms involved, variation in several traits is likely to have consequences for ecosystem functions. For example, leaf-shredding in *G. roeselii* strongly depends on body size, which varied in dependence of several ecological parameters.

**Electronic supplementary material:**

The online version of this article (10.1186/s12983-019-0327-8) contains supplementary material, which is available to authorized users.

## Background

### Selective landscapes along complex stream gradients

Environmental gradients provide a unique opportunity to evaluate the relative importance of various forms of both natural [1, 2] and sexual selection [3, 4] for creating intraspecific phenotypic variation [1–7]. Changes of environmental conditions can be accompanied by divergence of phenotypic traits when it allows local populations to reach a new fitness peak within a spatially heterogeneous selective landscape [6, 8]. Studies focusing on environmental gradients acknowledge the multitude of different ecological gradients and described, for instance, gradual variation of abiotic selection factors across latitudinal [9–11], or altitudinal [12] gradients, as well as gradients formed by environmental stressors like temperature [13], salinity [14, 15], or acidification [16, 17]. Other studies focused on biotic selection factors such as predation [18], as well as intra- [19] or inter-specific competition [20]. Beside spatial variation, selection pressures can also vary substantially across time [21, 22].

Streams and the associated limnic ecosystems are shaped by a widespread form of an environmental gradient in which an array of abiotic and biotic selection factors vary systematically in space and time, e.g., from source regions over smaller tributaries to slow-flowing lowland river sections [23–25] or between seasons [21, 23]. Predictable longitudinal changes include, amongst others, changes in primary carbon sources, with allochthonous organic material typically representing the main food source for headwater communities and autotrophic primary production becoming more important at downstream sites [23, 25]. Accumulation of various forms of nutrients usually results in a higher nutrient availability in downstream sections of rivers [23], while in anthropogenically transformed landscapes also pollutants tend to accumulate in downstream sections (e.g., pharmaceuticals [26], pesticides [27], or plastics [28]). Temporal changes in different river sections include, amongst others, flow regimes and water temperatures: source habitats usually show little seasonal fluctuation with respect to abiotic conditions—such as water temperatures [29]—and closely mirror the conditions seen in the groundwater bodies feeding them [23]. The stability of abiotic conditions rapidly decreases with increasing distance from the source(s), especially when the stream is no longer covered by forest canopy, such that water temperatures will attain greater variance due to solar input [25, 30]. These river sections often experience pronounced fluctuations in abiotic conditions, as they undergo recurrent catastrophic flooding (e.g., after snow melt [31]), or temporal desiccation either during hot/dry seasons [32, 33] or as a result of climate change [34]. Abiotic conditions reattain a more stable state in downstream river portions, where multiple tributaries interconnect to form an extensive wetland system [23, 25].

Despite the generality of some of these characteristics of river gradients at a larger geographical scale [23–25], the situation in reality tends to be more complex, not only because numerous landscape features alter the hydrogeological setting at a local scale [35, 36], but also because several forms of anthropogenic stressors [37, 38] contribute to the complexity of selective landscapes along river gradients [23–25]. Obviously, it is close to impossible to empirically capture all abiotic and biotic components characterizing a given stream gradient. Moreover, several factors—including those that were empirically assessed and those not assessed—can be tightly interrelated (e.g., oxygen content and temperature, or the occurrence of certain pollutants [39]). Empirical studies reporting intraspecific trait divergence along stream gradients and ascribing this divergence to one particular component of the gradient are, therefore, prone to overlooking that divergence may have been driven instead by another, interrelated selection factor, or by multiple factors [6, 40].

### Using amphipods to study phenotypic differentiation along stream gradients

Our present study, for the first time, reports intraspecific differentiation of morphological and life-history phenotypes along stream gradients in a freshwater amphipod, *Gammarus roeselii* Gervais 1835 [41]. Given their small size and benthic life-style [42, 43], amphipods have a low motility compared to, e.g., teleost fishes—which are frequently used in studies on local adaptation along stream gradients [5, 44, 45]—potentially rendering members of this group prime candidates to study adaptive diversification (i.e., evolutionary divergence or adaptive plasticity; see Additional file 1: Material S1 for additional information) over small geographic scales. We evaluated the roles of both natural selection (e.g., low oxygen levels favouring increased gill surface areas [46]; Table 1) and sexual selection for driving divergence in multiple trait suites. In the latter case, divergent selection could arise as an indirect consequence of local differences in population densities (a correlate of mate availability [75]), sex ratios [59, 76], or the potential for mate competition [59, 77] (Table 1). Nevertheless, the involvement of sexual selection in creating intraspecific phenotypic variation along stream gradients remains understudied [3, 78, 79].

**Table 1:** Phenotypic traits assessed in our present study and (unidirectional) predictions for trait divergence along single components of stream gradients

Category	Phenotypic trait	Affected by components of the river gradient	Rationale
Body size and condition-related adult traits	Body length	▼Temperature [47, 48], [49, 50]*	Increased body size may translate into an enhanced tolerance to low temperatures.
		▲Resources [51, 52]	Increased resource availability may result in larger adult body size, which is often correlated with increased investment into reproduction [53].
		▼Oxygen [48]	Oxygen availability is coupled with temperature regimes and probably a major mechanistic determinant of growth and general development.
		▲Competition [54]*	Large specimens are more competitive at high conspecific densities.
		▼ (Micro)pollution [55]*	Reduced size below sewage treatment works can be a result of the water containing endocrine-disrupting chemicals.
		▼Predation [56–58]	Relatively larger individuals experience higher predation risk than smaller ones.
		▲Sexual selection [59]*, [60]	Male pairing success is positively related to body size.
	Body weight (size-corrected)	▲ Resources [61]*	Higher resource availability (usually after leaf fall in autumn and winter) results in increased body condition.
		▼Competition [53]	Intraspecific competition results in fewer resources being available per individual to invest into somatic maintenance and reproduction.
		▼ Predation [62]*, [63]	Predator cues can induce behavioural alterations (e.g., reduced foraging), resulting in lower body condition.
Offspring-related phenotypic traits	Fecundity (number of offspring per brood)	▲ Resources [58]	Higher resource availability allows for more investment into egg production.
		▲ Predation [57]*	Predators increase extrinsic mortality, favouring *r*-selected phenotypes with more, but smaller embryos (see also *Embryo size*).
		▼ Pollution [64]	Pollution (sewage and heavy metals) derived from industrial and domestic sources reduce fecundity.
		▼ (Micro)pollution [65]*	Endocrine-disrupting chemicals cause intersexuality in amphipods, leading to a reduced fecundity.
	Embryo size	▼Temperature [50, 66, 67]*	Larger embryo size during winter may be driven by a higher tolerance to low water temperatures. Absence of cold temperatures in thermally-polluted streams reduces selection for large embryo size.
		▼ Resources [68]*	Under high resource availability embryo size can be reduced, while embryo size should be increased under resource shortage.
		▼ Predation [57]*	The optimal egg size depends on the relationship between juvenile survival and egg size [57]*. The trade-off between offspring size and fecundity [69]* allows females to increase fecundity by producing a few smaller embryos. Smaller size (allowing higher fecundity) is only beneficial if enough offspring survive.
Physiological traits	Gill surface area	▼ Pollution [70]*	Toxic metals are taken up by aquatic crustaceans via the gills. Hence, increased gill area might be disadvantageous under elevated heavy metal concentrations.
		▼ Oxygen [46]	High oxygen supply allows species to have smaller gill areas.
Traits used for intrasexual communication and mate defense	Antennae length	▲Sexual selection [60, 71]*	Male antennae are important for locating and evaluating potential mates.
		▲ Male biased sex-ratio/intraspecific density	Sex ratios affect male mating behaviour [72] and, therefore, the strength of sexually selected male traits. Male-male competition should increase at high population densities and /or male biased sex-ratios because of the high encounter rate between competitors.
		▲ (Micro)pollutants [73]*, but see [74]*	Longer antennae were induced by exposure to non-ionic surfactant 4-nonylphenol [73]; no effect of estrogen 17 α-ethinylestradiol was observed [74].
	Gnathopod size	▲ Sexual selection [71]*	Male gnathopods play a central role in holding/securing the female before and during copulation (amplexus).
		▲ Male biased sex-ratio [72]*	Under male biased sex-ratios, male-male competition increases and males guard females longer.

We provide *a priori* predictions for the direction of evolutionary and/or plastic trait divergence in *G. roeselii* by agents of natural and sexual selection based on a literature survey including other amphipods (marked by asterisks [*]) and freshwater invertebrates in general. ▲Predicted positive association (increasing phenotypic trait values correspond with increasing values of the respective variable); ▼predicted negative association

Acknowledging the complexity of selective regimes in stream gradients [23–25, 37, 38], where multiple selective forces may have complex synergistic, non-additive, or antagonistic effects [80] (potentially overriding certain evolutionary responses, e.g. [81]), we refrain from providing unidirectional predictions for the different trait suites studied herein; an overview of potential predictions can be found in Table 1. We considered physiological traits that are likely to be chiefly under natural selection from abiotic agents of selection (e.g., gill surface areas should vary in dependence of local temperature and oxygen regimes [46]), reproductive life history traits known to be under natural selection from both abiotic and biotic selection factors (brood size/fecundity and offspring size [18, 82, 83]), and traits that are primarily under sexual selection (e.g., size of the male gnathopods, which are involved in mate securing and defense [84]; Table 1).

### Intraspecific phenotypic differentiation in invasive amphipods

In our present study we examined a non-native species that reached Central Europe approximately 150 years ago [41] (see Additional file 1: Material S2 for additional information). While the examination of a species that colonized Central Europe relatively recently already drew the focus of our study towards questions related to contemporary evolution [85, 86] or adaptive phenotypic plasticity [87], we included yet another level of questions related to rapid evolutionary change/adaptive plasticity and compared streams with and without anthropogenic thermal pollution (starting approximately 50 years ago [88, 89]). Studying biotic responses to thermal pollution has become increasingly popular to forecast the potential effects of global warming [90]. Thermal pollution should primarily affect naturally selected traits (e.g., via lower oxygen availability), but could (mediated, e.g., by longer reproductive seasons) also affect the strength and direction of sexual selection (Table 1). Given that studies on inter- and intra-specific body size variation have been at the forefront of research on climate-related evolution for centuries (Bergman’s rule, originally formulated for endotherms [91] but partly also applied to ectotherms [47]), we assessed body size as another dependent variable (Table 1).

**Fig. 1:**
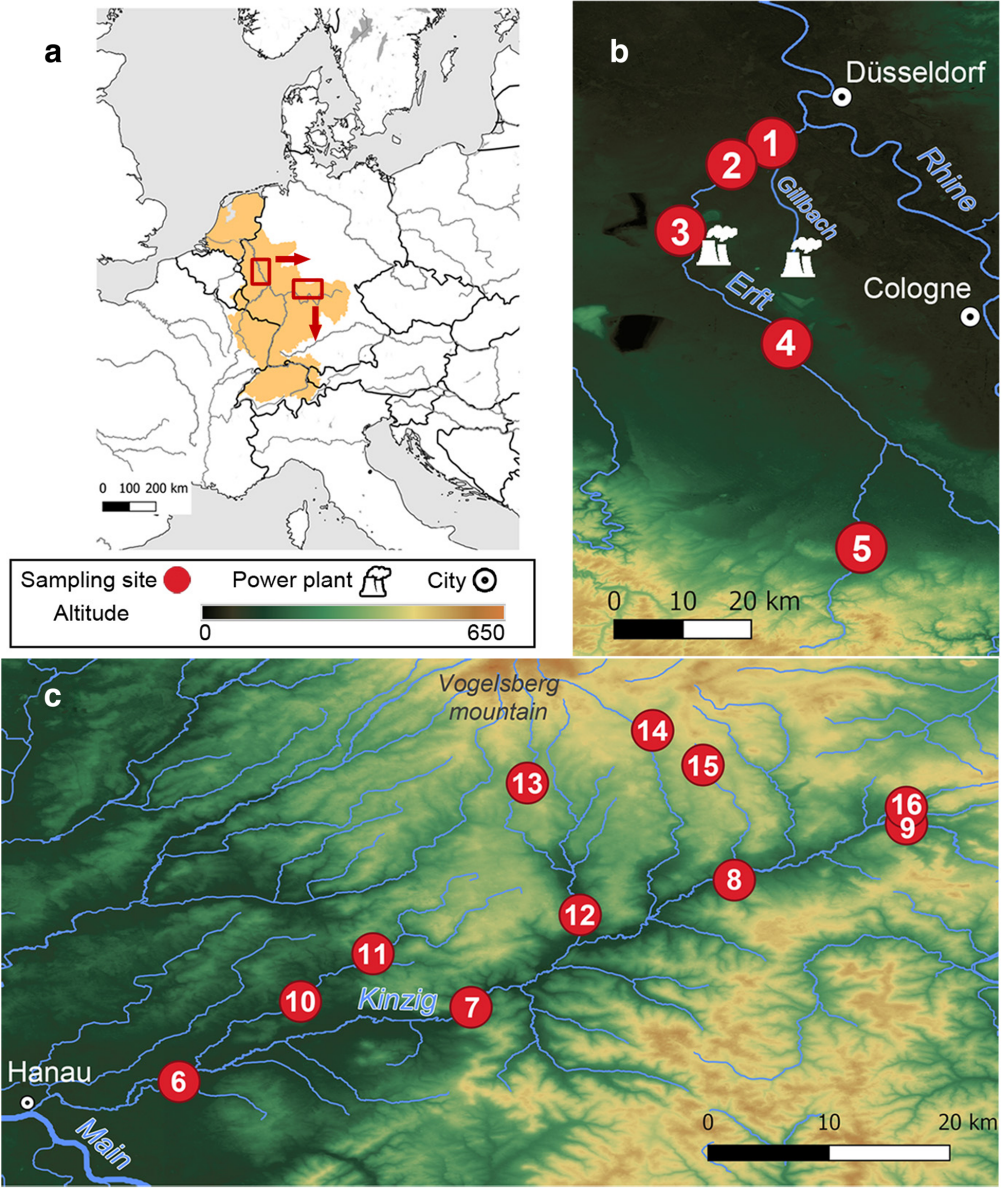
Map of our study areas. **a** Location of the Rhine catchment in Central Europe with the two studied tributaries: **b** Erft, which receives thermal pollution in the form of cooling water from several coal power plants and **c** Kinzig, which does not receive artificially heated cooling water. Locations and number codes of our 16 sampling sites are indicated (maps created with QGIS 3.4.2; the altitude layer was retrieved from https://lpdaac.usgs.gov)

Our study was motivated by the idea that freshwater amphipods—including invasive species or those that expanded their distribution ranges relatively recently—could be established as a future model for several questions in evolutionary ecology, especially questions related to local adaptation [6, 8], contemporary evolution [85] or adaptive phenotypic plasticity [87], life-history evolution [18, 82], and climate change biology [92]. Our present study is centred on two major questions:

(1) Do we find small-scale phenotypic differentiation in non-native *G. roeselii* approximately 150 years upon their arrival in Central Europe [41] along repeated river gradients? To answer this question, we sampled amphipods from 16 sites along two streams in Germany (Fig. 1) and assessed an array of phenotypic traits (Table 1).

(2) If phenotypic differentiation is uncovered, can we ascribe at least parts of this variation to known drivers of both natural and sexual selection processes (or triggers of adaptive plasticity), including selection factors arising from anthropogenic activities (Table 1)?

## Methods

### Study species and sample collection

We focused on a ‘naturalised’ [93] non-native species, *Gammarus roeselii*, which has spread throughout Central Europe and established stable populations since the nineteenth century [41]. *Gammarus roeselii* is usually considered as a downstream-adapted species [94, 95]. However, several populations in downstream sections of Central European streams were recently replaced by the more recent invaders *Dikerogammarus villosus* and *Echinogammarus ischnus* [96–98]. On the other hand, *G. roeselii* is nowadays found in at least some Central European first order streams at altitudes above 400 m (this study).

We collected specimens in two subcatchments within the Rhine catchment (Fig. 1). The Erft is located in a heavily exploited, urbanized area and in downstream sections receives cooling water from several nearby coal power plants [88, 89], which significantly increases average water temperatures (mean ± SD during winter: 6.1 ± 1.1 °C at thermally non-polluted sites and 12.4 ± 1.2 °C at thermally polluted sites; during summer: 17.5 ± 3.0 °C at thermally non-polluted sites and 23.7 ± 0.3 °C at thermally polluted sites), while our sampling sites in the Kinzig are located within a long-term ecological research area (LTER [99]), and its tributaries do not receive thermally polluted water (Additional file 1: Table S1). We caught amphipods using a multi-habitat kick-sampling method [100]. To account for potential seasonal variation of the traits considered here, we sampled all sites twice, once in February (winter sampling) and once in May of 2017 (summer sampling). All captured specimens were immediately preserved in 96% ethanol until they were processed in the laboratory. As *G. roeselii* has been reported to occasionally co-occur with the congeners *G. fossarum* and *G. pulex* [94, 101], we verified species identity of all specimens under a stereomicroscope (Olympus SZX12 with an Olympus DF PLFL 0.5X PF objective) using standard identification keys [102, 103]. Altogether, we dissected *n* = 1648 *G. roeselii* from 16 sampling sites (*n* = 373 from thermally polluted and *n* = 1275 from thermally non-polluted sites; Additional file 1: Table S3).

### Environmental parameters

We assessed a range of environmental variables that are frequently used to characterize stream gradients [23–25] and show gradual variation along the examined river stretches (Additional file 1: Table S1). At each site, we measured water temperature [°C] (using a WTW Multi 340i, TetraCon® 325), flow velocity [m s^− 1^] using a portable flowmeter (P770, Dostmann electronic GmbH), oxygen content [mg L^− 1^] (WTW Multi 340i, CellOx® 325), and conductivity [μS cm^− 1^] (WTW Multi 340i, TetraCon® 325). We estimated average stream depth at our sampling sites as falling into one of three categories (< 0.5 m, 0.5–1 m, > 1 m). Similarly, we categorized average stream width as < 7 m, 7–12 m, or > 13 m. Altitudinal information [m above sea level] was extracted from Google Earth (http://earth.google.com/). We estimated conspecific population densities by counting all specimens caught by two persons that applied multi-habitat kick sampling [100] for 30 min in a river stretch of 50 m. We categorized population densities as low (0–50 individuals), medium (51–200 individuals), or high (> 200 individuals). Based on the obtained sample of *G. roeselii* specimens, we calculated sex-ratios (number of females/number of males) for each sampling site. Since several explanatory variables showed strong intercorrelations (Table 2), we applied a factor reduction (PCA) using the Varimax option (based on the Kaiser normalization rotation method [104]) as implemented in SPSS 23. The four resulting rotated principal components (henceforth called ‘environmental PCs’) with eigenvalues > 1.0 were used as explanatory variables for all further analyses (for axis loadings see Table 2). Cumulatively, they explained 71.8% of the total variation (PC 1 explained 32.8%, PC 2: 14.5%, PC 3: 12.9% and PC4 11.5%).

**Table 2:** Results of a factor reduction procedure (PCA) on 11 environmental parameters measured at our 16 sampling sites

	Env. PC 1	Env. PC 2	Env. PC 3	Env. PC 4
Stream width [m]	**0.89**	−0.15	− 0.01	− 0.02
Stream depth [m]	**0.79**	−0.32	0.22	−0.04
Water temperature [°C]	0.39	0.01	**0.58**	0.49
Oxygen content [mg L^−1^]	0.23	0.10	**−0.85**	0.20
Flow velocity [m s^−1^]	0.06	**0.59**	0.01	−0.19
Conductivity [μS cm^−1^]	**0.80**	0.27	−0.21	0.14
Density (catch-per-unit effort)	0.20	**0.61**	0.40	0.23
Altitude [m]	**−0.83**	−0.16	0.15	0.05
Sex ratio (females/ males)	−0.06	**0.77**	−0.24	0.01
Thermal pollution (yes/ no)	**0.79**	0.22	0.14	0.11
Season (summer/ winter)	−0.04	−0.10	−0.10	**0.93**

Shown are PC axes (‘environmental PCs’) with eigenvalues > 1.0; axes were varimax-rotated using the Kaiser Normalization method. Variables with |axis loading| ≥ 0.5 are highlighted in bold font

### Life history and morphometric data

We collected information on male and female life-histories (see Additional file 1: Material S3 for additional information) and morphological traits from 50 to 128 individuals per population (Additional file 1: Table S3). All measurements of distances or areas were conducted under a stereomicroscope (OLYMPUS SZX12) with an OLYMPUS DF PLFL 0.5X PF objective and an attached OLYMPUS SC30 camera connected to a computer. We used the software Cell^A (Olympus) for all linear and area measurements.

#### Sexing, adult body size and weight

**Fig. 2:**
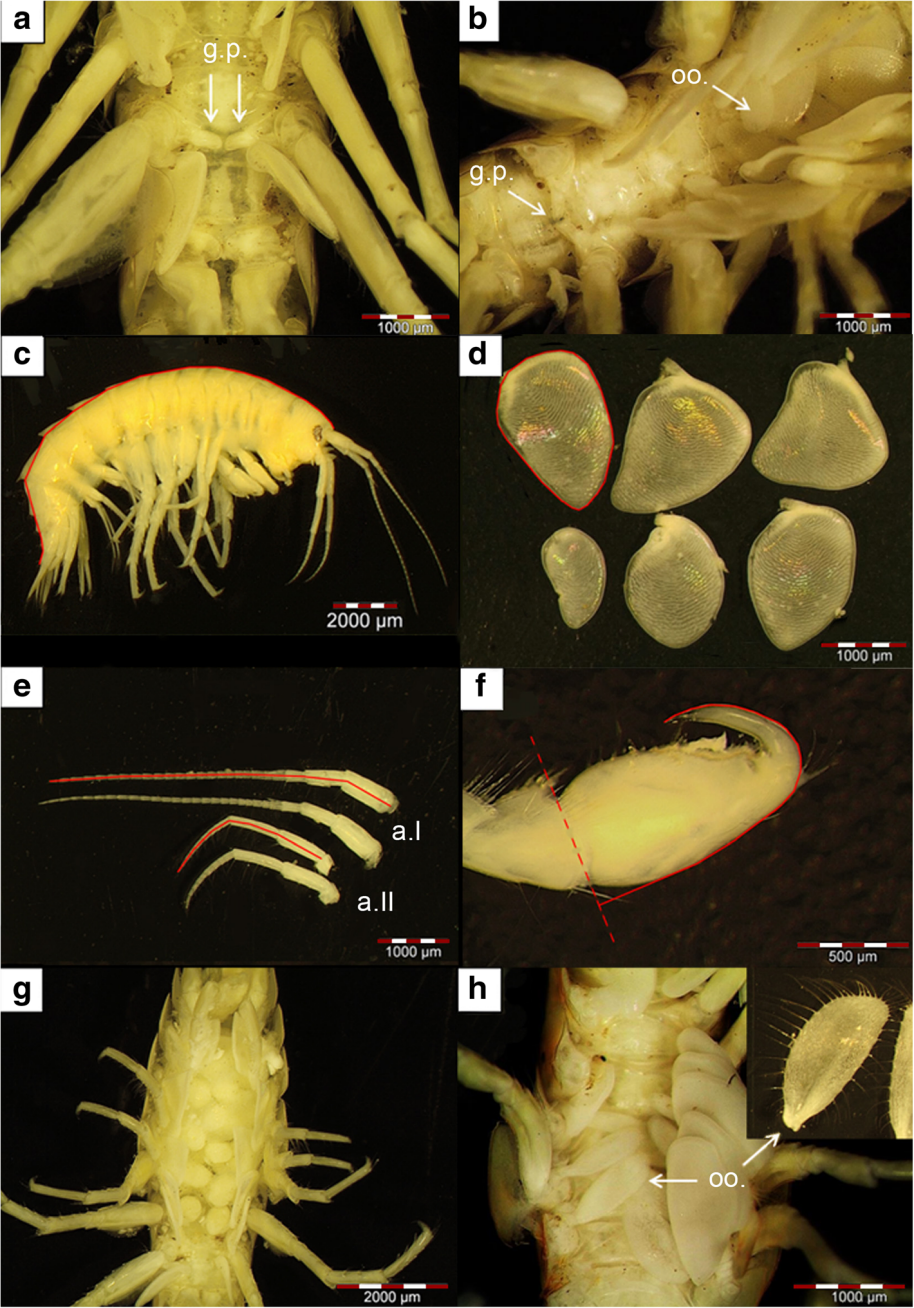
Overview of morphological characteristics assessed in this study. **a** Males were identified by the presence of genital papillae (g.p.). **b** Intersexual individuals show female (oostegites; oo.) and male (genital papillae; g.p.) sexual characteristics. The following parameters we measured as distances or areas (marked by red lines) and used to assess population variation in **c** body length, **d** gill area (circumference of the six gills on the right body site; considered herein as a ‘physiological trait’), **e** lengths of the 1^st^ antennae (a.I) and the smaller 2^nd^ antennae (a.II), from the first pedunculus to the tip of the flagellum, and **f** size of the 1^st^ gnathopod on the right body size of males (length from the tip of the dactylus to the base of the propodus). **g** Females carry their developing broods in an external brood pouch that is formed by (**h**) four pairs of oostegites (oo.)

Specimens were sexed according to external sexual characteristics: males were identified by the presence of genital papillae (Fig. 2a) which lie ventrally in the middle of the bases of the 5th pereopods [103]. Female gammaridean amphipods usually have four pairs of oostegites between pereopods 2–6 [105]. In some cases, intersexual individuals [106] with both genital papillae and oostegites were observed (Fig. 2b; see Additional file 1: Material S4 for additional information and a posteriori analyses on data from our present study).

We determined each specimen’s body length [mm] from the tip of the rostrum to the telson tip (Fig. 2c). To do so, alcohol-stored specimens were placed in lateral position in a wax-filled Petri dish and were carefully straightened using preparation needles. We ensured that all specimens were similarly stretched before measuring their body length. We also measured head capsule length as an alternative proxy for body size [107]. However, both traits were strongly collinear (Pearson’s *r* = 0.92; Additional file 1: Figure S1), and so we used body length throughout. Before we assessed somatic dry weight [mg], we removed all eggs from the female marsupium, after which we dried specimens for 24 h at 60 °C in a drying oven (Heraeus Type B5042). We stored dried individuals in a desiccator containing dry silica gel to prevent water-uptake while cooling down to room temperature. Afterwards, we weighted specimens to the nearest 0.1 mg on a micro-scale (SI-234 Denver Instrument).

#### Physiological traits

We additionally measured gill surface areas. Gills are a multi-functional organ, involved in several physiological processes, tightly linked to physiological homeostasis of oxygen/CO_2_ concentrations [108], osmoregulatory ion transport [109] and excretion of nitrogenous waste products [109]. Furthermore, toxic metals are taken up by aquatic crustaceans via the gills, such that the gills play an important role in mediating the response of aquatic amphipods to contaminants [109, 110]. Dissecting the delicate gills of amphipods is a tedious and time-consuming task. We, therefore, decided to concentrate on one sex and measured gill surface areas in males only, even if this introduces a potential bias and prevented us from detecting potential sex-specific variation.

We placed males in a dorsal position onto agarose gel and fixed them with two fine needles, upon which we carefully removed the gills from the right body site at their base using a fine preparation needle. We photographed the gills and measured their circumference (Fig. 2d). The area of each gill was determined using the image analysis software Cell^A (Olympus). We summed data from all six gills per individual as a proxy of the respiratory surface area [mm^2^]. However, our method did not allow us to detect potential changes in secondary and tertiary lamella [111], which might be an alternative phenotypic response to increase the overall respiratory surface area.

#### Traits used for inter-sexual communication and mate guarding

Male gammarids use their antennae during mate assessment [60, 71]. Accordingly, their antennae tend to be sexually dimorphic [112], and the length of the antennae in gammarids [71, 113] and other crustaceans [60] can show variation between populations. We carefully removed both pairs of antennae of all individuals (males and females) at their base using a fine preparation needle. Antenna length [mm] was assessed by measuring the distance from the base of the first pedunculus to the tip of the flagellum (Fig. 2e). We calculated mean values from the antennae on the left and right body sides.

Gnathopods play a central role for holding/securing the female before and during copulation (called amplexus [105]), which includes defending the female from rivals that attempt to take over the female [72, 84]. To assess potential population variation in this trait [113], we measured the size [mm] of the 1^st^ gnathopod on the right body side in all males. To do so, the gnathopod was carefully removed and photographed. We drew a virtual orientation line at the base of the propodus and measured the distance between the base of the propodus to the tip of the dactylus (Fig. 2f).

#### Offspring-related phenotypic traits

**Fig. 3:**
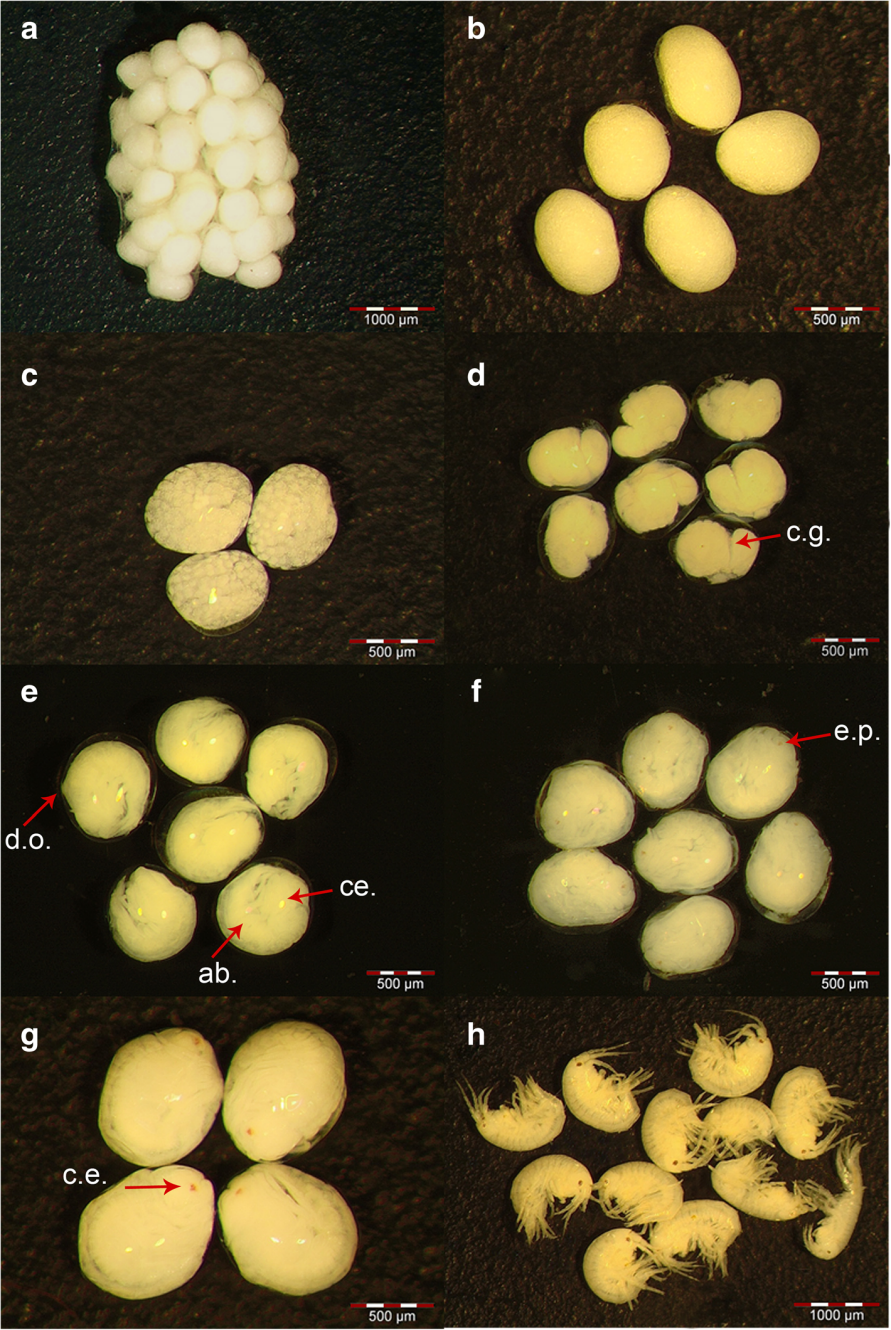
Stages of embryonic development in *G. roeselii*. **a** Newly fertilized eggs (stage 1), surrounded by a membranous sac. **b** Detailed view of eggs at stage 1 with no visible cell cleavage. **c** Stage 2 is characterized by the formation of large yolk cells through holoblastic cell cleavage. **d** Embryos at stage 3 show a caudal groove (c.g.). **e** At stage 4, the embryo’s body is separated into cephalothorax (ce.) and abdomen (ab.). The dorsal organ (d.o.) is visible. Embryos show a comma-like body shape and body appendages start to develop. **f** Red eye pigmentation (e.p.) and fully developed body appendages are characteristic of developmental stage 5. **g** Fully developed juveniles with completely developed compound eyes (c.e.) that are still surrounded by the external chorion of the egg indicate developmental stage 6. **h** Newly hatched juveniles (stage 7) have ruptured and left the chorion

For each female, we determined fecundity by carefully removing all eggs from the brood pouch (the marsupium [66, 105]; Fig. 2g) with a fine brush and counting them. Data from females harbouring stage 7 embryos (see below) were excluded, as some of the juveniles may already have left the marsupium. To estimate egg volumes [mm^3^], we measured the longest and shortest axis of each egg in 2D view and used an ellipsoid formula to approximate egg volume (see [67]). We identified embryonic developmental stages according to earlier descriptions for *Gammarus* spp. [67, 108, 114]. A detailed description can be found in Additional file 1: Material S5, and exemplary photos are shown in Fig. 3.

### Statistical analyses

We asked whether and how environmental parameters used to characterize stream gradients and condensed into principal components (Table 2) drive divergence of multiple phenotypic traits. To this end, we applied generalized least squares models (GLS) using the *gls*-function, implemented in the *nlme* package in R [115] fitted by maximum-likelihood estimation and assuming a Gaussian error distribution. To account for spatial autocorrelation, we fit our GLS models with an autoregressive (AR-1) correlation structure [116]. Each of the following variables was modelled separately: ‘body length’, ‘dry weight’, ‘length of 1^st^ antennae’, and ‘length of 2^nd^ antennae’. For females, we additionally analysed ‘fecundity’ and ‘egg volume’ and for males ‘gill area’ and ‘gnathopod size’. We included the explanatory variables ‘environmental PCs 1 – 4’, ‘sex’ (in all cases where data were available from both sexes) and ‘body length’ to account for ontogenetic changes in trait values. For the model on egg volume, we additionally considered ‘developmental stage’ as an explanatory variable to account for ontogenetic changes in egg/embryo size during development. The statistical significance of explanatory variables in the linear models was evaluated by Type III ANOVAs (using the *Anova*-function implemented in the *car* package [117]). Assumptions of normality of residuals was assessed visually by inspecting QQ-plots and plots of residuals vs. fitted values (Additional file 1: Figure S3). In the case of significant effects, we calculated and visualized predicted marginal effects of environmental variables on dependent variables using the *ggeffects* package [118]. All statistical analyses were performed in R 3.5.1 [119].

## Results

Inspection of axis loadings of the four principal components (PCs) retained from our factor reduction of various environmental parameters (Table 2) allows the following interpretation: PC 1 reflected variation from upstream to downstream conditions, with higher PC-scores being associated with greater stream width, stream depth, conductivity and thermal pollution, while altitude had a negative axis loading. PC 2 captured variation in flow velocity, conspecific densities and sex ratios, whereby positive values represented fast-flowing sites with high densities and female-biased sex-ratios. PC 3 received high axis loadings from water temperatures and oxygen content and could be interpreted as describing the gradient from cold, well-oxygenated sites towards warmer sites with lower oxygen content. Finally, PC 4 captured seasonal variation between summer (negative values) and winter samplings (positive values). In the following, we will present significant outcomes of trait-wise generalized least squares models (Tables 3, 4, 5) in light of the information captured by the four PCs that served as explanatory variables.

**Table 3:** Results of generalized least squares models on body size/condition-related phenotypic and physiological traits of adult *G. roeselii*

	(a) Body length			(b) Dry weight			(c) Gill surface area		
	*df*	χ^2^	*p*	*df*	χ ^2^	*p*	*df*	χ ^2^	*p*
Body length (covariate)	**–**	**–**	**–**	**1**	**4524.10**	**< 0.001**	**1**	**2013.60**	**< 0.001**
Sex	**1**	**39.92**	**< 0.001**	**1**	0.12	0.73	**–**	**–**	**–**
Environmental PC 1	**1**	**23.92**	**< 0.001**	1	1.53	0.22	**1**	**15.20**	**< 0.001**
Sex × env. PC 1	**1**	< 0.001	0.98	**1**	2.10	0.15	**–**	**–**	**–**
Environmental PC 2	**1**	**4.00**	**0.046**	**1**	**9.89**	**0.002**	1	0.84	0.36
Sex × env. PC 2	1	1.08	0.30	1	1.77	0.18	**–**	**–**	**–**
Environmental PC 3	1	2.47	0.12	1	0.01	0.91	1	0.00	0.99
Sex × env. PC 3	1	1.87	0.17	1	0.99	0.32	**–**	**–**	**–**
Environmental PC 4	1	0.71	0.40	**1**	**6.33**	**0.012**	**1**	**10.37**	**0.001**
Sex × env. PC 4	1	0.09	0.77	1	1.72	0.19	**–**	**–**	**–**

Shown are the results of three independent models using (a) adult body size, (b) body weight, assessed after drying samples overnight (in both sexes) and (c) gill surface areas (only males) as the dependent variables. Environmental PCs (Table 2), as well as sex and body length (where applicable) were coded as independent (explanatory) variables. Significant effects are shown in bold

**Table 4:** Results of generalized least squares models on phenotypic traits used for mate assessment and mate defence

	(a) 1^st^ Antennae			(b) 2^nd^ Antennae			(c) Gnathopod length		
	*df*	χ^2^	*p*	*df*	χ^2^	*p*	*df*	χ^2^	*p*
Body length	**1**	**4388.92**	**< 0.001**	**1**	**4912.87**	**< 0.001**	1	0.66	0.42
Sex	**1**	**152.65**	**< 0.001**	**1**	**1381.67**	**< 0.001**	**–**	**–**	**–**
Environmental PC 1	**1**	**63.08**	**< 0.001**	**1**	**48.98**	**< 0.001**	1	0.01	0.91
Sex × env. PC 1	1	0.08	0.78	**1**	**6.64**	**0.010**	**–**	**–**	**–**
Environmental PC 2	1	0.35	0.55	1	2.89	0.089	1	0.71	0.40
Sex × env. PC 2	**1**	**5.66**	**0.017**	1	0.05	0.83	**–**	**–**	**–**
Environmental PC 3	**1**	**34.23**	**< 0.001**	**1**	**11.39**	**0.001**	1	0.33	0.57
Sex × env. PC 3	1	0.10	0.76	1	< 0.001	0.96	**–**	**–**	**–**
Environmental PC 4	**1**	**39.96**	**< 0.001**	**1**	**13.62**	**< 0.001**	1	< 0.001	0.98
Sex × env. PC 4	1	0.55	0.46	1	0.54	0.46	**–**	**–**	**–**

We assessed the following dependent variables: (a) length of the 1^st^ and (b) 2^nd^ antennae (which males use for mate detection and assessment) in both sexes. (c) Gnathopod size (used by males during precopulatory mate guarding, called amplexus) was assessed only in males. Environmental PCs (Table 2), body length and sex (where applicable) served as predictor variables. Significant effects are shown in bold

**Table 5:** Results of generalized least squares models on reproductive life-history traits in females

	(a) Fecundity			(b) Egg size		
	*df*	χ^2^	*p*	*df*	χ^2^	*p*
Female body length	**1**	**207.72**	**< 0.001**	**1**	**13.56**	**< 0.001**
Developmental stage	**–**	**–**	**–**	**1**	**1784.89**	**< 0.001**
Environmental PC 1	**1**	**20.08**	**< 0.001**	1	1.47	0.23
Environmental PC 2	**1**	**78.20**	**< 0.001**	1	3.05	0.081
Environmental PC 3	1	2.95	0.086	1	0.00	0.98
Environmental PC 4	1	1.43	0.23	1	0.02	0.89

Two independent models considered (a) egg number per brood (fecundity) and (b) egg size as the dependent variables, while environmental PCs (Table 2), body length and developmental stage (where applicable) served as predictor variables. Significant effects are shown in bold.

### Body size and condition-related adult traits

**Fig. 4:**
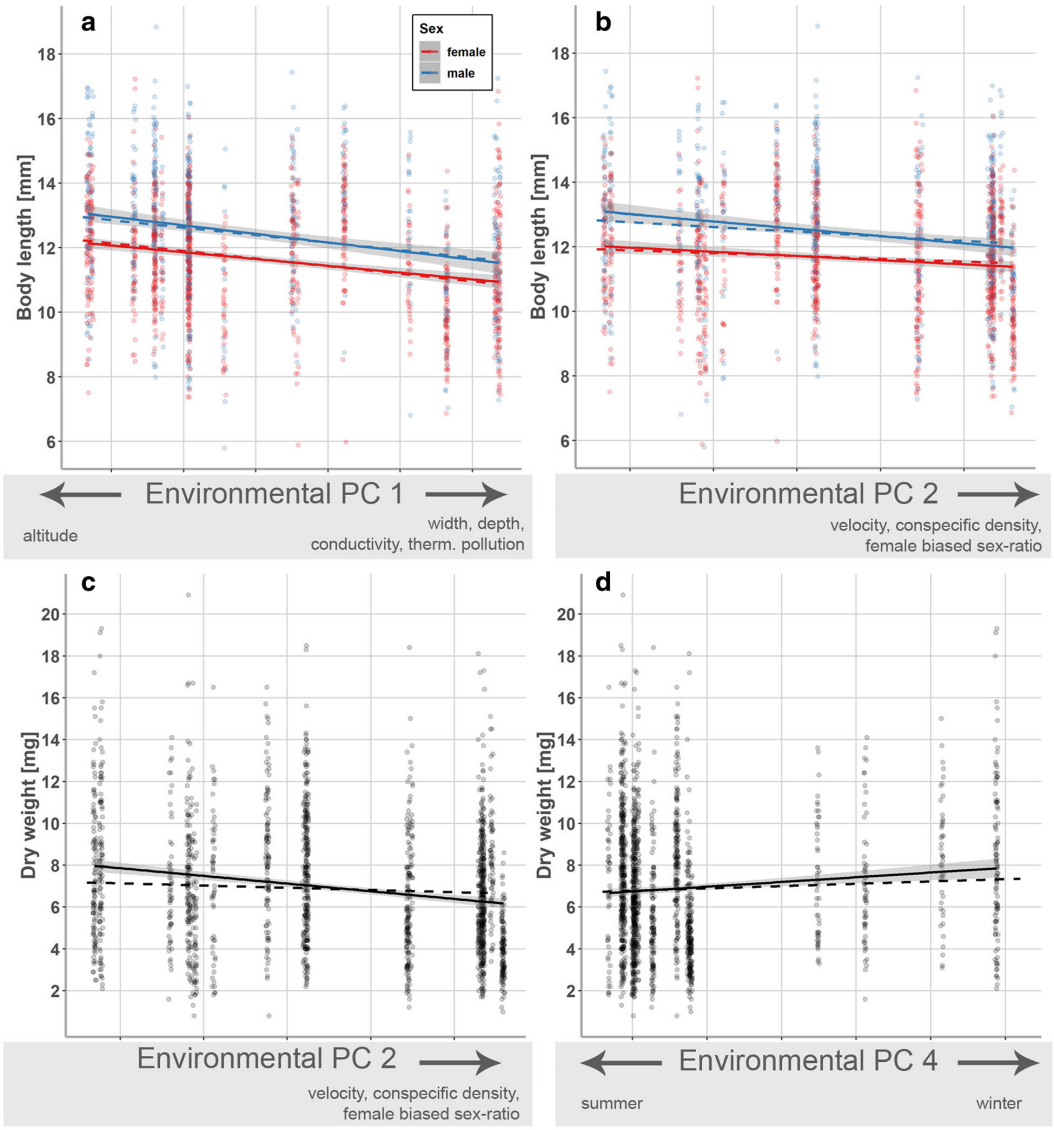
Visualization of significant model terms in generalized least squares models on body size/condition-related adult phenotypic traits. Principal components (PCs) capturing variation in environmental parameters along the examined stream gradients (Table 2) served as explanatory variables. Shown are effects for **a**, **b** body length, and **c**, **d** body weight (dry weight), which are presented separately for both sexes if the models uncovered significant sex-effects (Table 3). Solid lines show linear relationships of raw data with 95% confidence intervals (shaded area), while dashed lines represent the linear relationships based on predicted values that were adjusted for other predictors in each model. For display purpose, data points were slightly shifted using the *position_jitter*-function in ggplot2

Adult body size decreased significantly from upstream to downstream sites (i.e., along environmental PC 1; χ^2^ = 23.92, *p* < 0.001; Table 3a; Fig. 4a) and with increasing conspecific densities (PC 2; χ^2^ = 4.0, *p* = 0.046; Fig. 4b). Likewise, adult body weight decreased significantly along environmental PC 2 (χ^2^ = 9.89, *p* = 0.002; Table 3b; Fig. 4c) and showed seasonal fluctuation, with greater body weight being observed in winter (PC 4; χ^2^ = 6.33, *p* = 0.012; Fig. 4d).

### Physiological traits

**Fig. 5:**
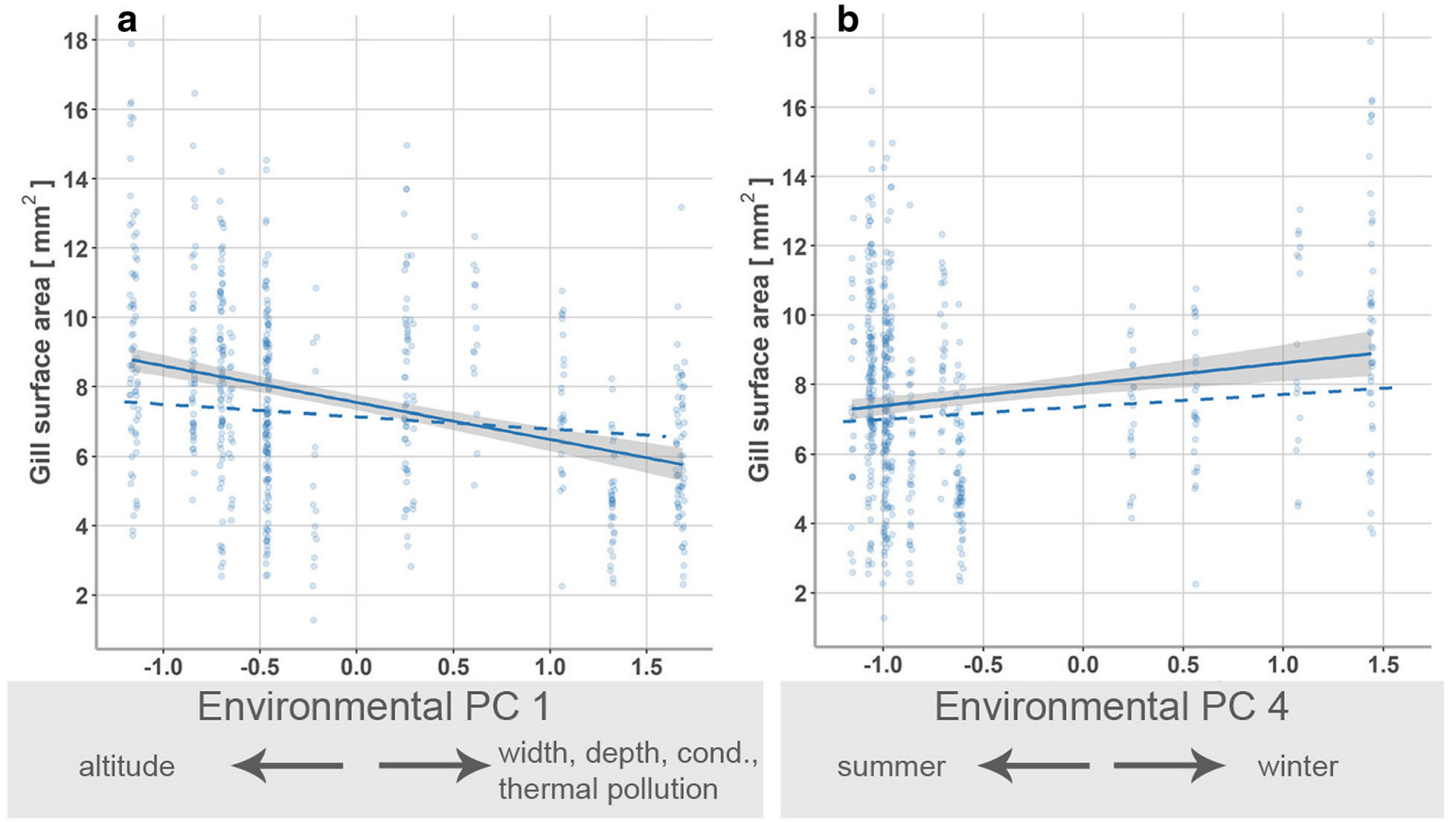
Visualization of significant model terms in generalized least squares models on gill surface areas. Shown are the relationships between male gill surface areas and **a** environmental principal component (PC) 1 and **b** PC 4 (Table 3). Solid lines show linear relationships of raw data with 95% confidence intervals (shaded area), while dashed lines represent the linear relationships based on predicted values that were adjusted for other predictors in the model (e.g., body length). For display purpose, data points were slightly shifted using the *position_jitter*-function in ggplot2

Gill surface areas decreased from upstream to downstream conditions (environmental PC 1; χ^2^ = 15.20, *p* < 0.001; Table 3c; Fig. 5a). Moreover, gills surface areas were greater during winter than summer (PC 4; χ^2^ = 10.37, *p* = 0.001; Fig. 5b).

### Traits involved in intersexual communication and mate guarding

**Fig. 6:**
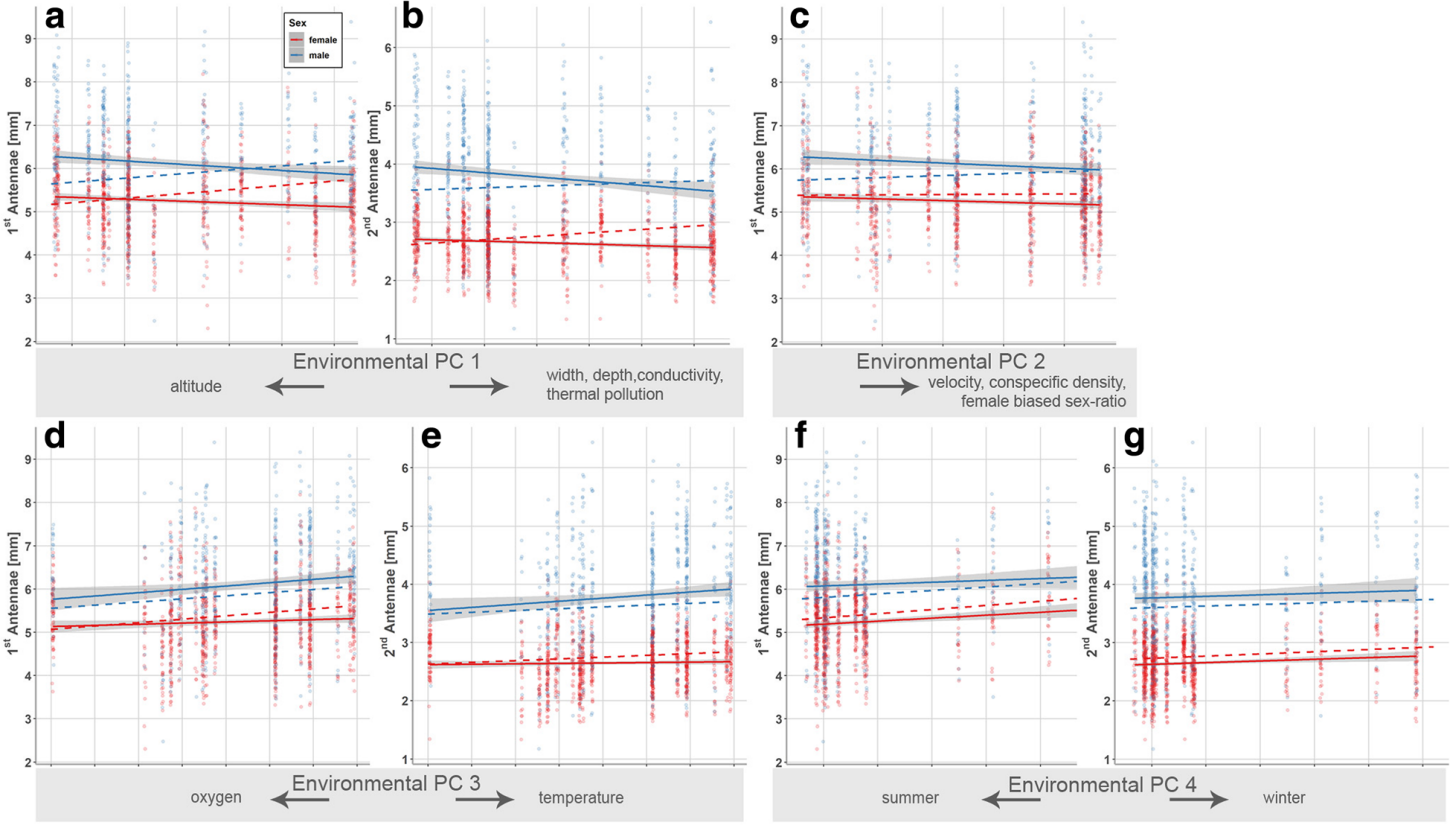
Visualization of significant model terms in generalized least squares models on phenotypic traits involved in mate finding and assessment. We depict marginal effects for gradual variation in the length of the 1^st^ (**a**, **c**, **d**, **f**) and 2^nd^ antennae (**b**, **e**, **g**) along environmental PC 1 – PC 4 (Table 4). Data are shown separately for both sexes if the models uncovered significant sex-effects. Solid lines show linear relationships of raw data with 95% confidence intervals (shaded area), while dashed lines represent the linear relationships based on predicted values that were adjusted for other predictors in each model (e.g., body length). For display purpose, data points were slightly shifted using the *position_jitter*-function in ggplot2

We found a pronounced sexual dimorphism in the length of the 1^st^ (χ^2^ = 152.65, *p* < 0.001) and 2^nd^ antennae (χ^2^ = 1381.67, *p* < 0.001; Table 4), with males possessing longer antennae than females. Moreover, antennae length showed gradual variation along all four environmental PCs (see significant effects of PC 1 – PC 4; Table 4). Specifically, both the 1^st^ (χ^2^ = 63.08, *p* < 0.001; Fig. 6a) and 2^nd^ antennae (χ^2^ = 48.98, *p* < 0.001; Fig. 6b) became longer along environmental PC 1. The significant interaction term of ‘sex × environmental PC 2’ reflects that the length of the 1^st^ antennae of males increased to a greater extent along PC 2 (i.e., with increasing population densities and increasingly female-biased sex ratios) than those of females (χ^2^ = 5.66, *p* = 0.017; Fig. 6c). Both antennae increased along environmental PC 3 (1^st^ antennae: χ^2^ = 34.23, *p* < 0.001; Fig. 6d; 2^nd^ antennae: χ^2^ = 11.39, *p* = 0.001; Fig. 6e) and were longer during winter compared to our summer sampling (PC 4; 1^st^ antennae: χ^2^ = 39.96, *p* < 0.001; Fig. 6f; 2^nd^ antennae: χ^2^ = 13.62, *p* = 0.001; Fig. 6g). Notably, we did not find significant divergence in male gnathopod length along environmental gradients (Table 4c).

### Reproductive characteristics

**Fig. 7:**
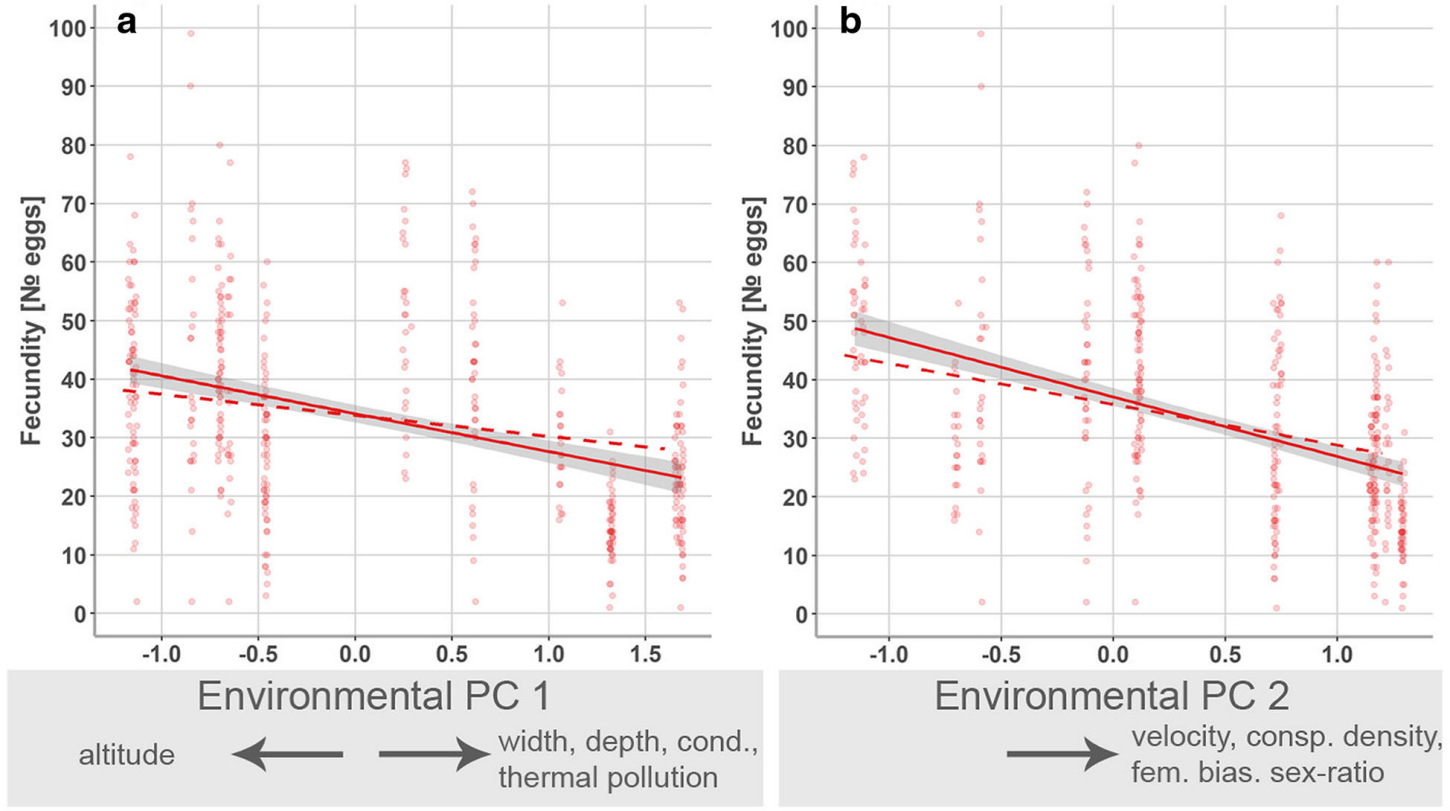
Visualization of significant model terms in generalized least squares models using offspring-related phenotypic traits as the dependent variable. We depict predicted marginal effects for female fecundity along principal components that contain information of environmental variation along the examined stream gradients [(**a**) environmental PC 1, (**b**) environmental PC 2; Table 5]. Solid lines show linear relationships of raw data with 95% confidence intervals (shaded area), while dashed lines represent the linear relationships based on predicted values that were adjusted for other predictors in each model. For display purpose, data points were slightly shifted using the *position_jitter*-function in ggplot2

Female fecundity (numbers of eggs per brood)—size-corrected by inclusion of the covariate ‘female body length’ (Table 5a)—decreased significantly from up- to downstream sites (i.e., along environmental PC 1; χ^2^ = 20.08, *p* < 0.001; Fig. 7a) and with higher conspecific densities (PC 2; χ^2^ = 78.20, *p* < 0.001; Fig. 7b). Egg size did not show gradual variation along any of the components of the stream gradient (Table 5b).

## Discussion

We found phenotypic differentiation among populations of *G. roeselii* occurring along the two river stretches examined in this study, including body size and weight, physiological traits (gill surface areas), reproductive life-history traits, and sexually selected morphological traits. Our study does not allow inferences regarding the relative roles of evolved differences (local adaptation) and phenotypic plasticity (Additional file 1: Material S1). Still, our analyses suggest that spatial variation in ecological conditions along the examined stream gradients drives the observed intraspecific differentiation over small geographic scales (i.e., within and between river catchments).

### Ecological significance of phenotypic differentiation

What are the potential ecological implications of our findings? Recent years have seen a steep increase in numbers of publications that either analyse empirical data (e.g., [120]) and/or use mathematical modelling (e.g., [121]) to understand ecological phenomena over large geographic scales (macroecology [122]). These approaches, however, usually treat species as “evolutionarily inert” entities with fixed (species-specific) ecological traits affecting ecosystem processes like nutrient and energy flow [123–125]. Nevertheless, both theoretical considerations and empirical evidence suggest that intraspecific phenotypic variation can affect ecosystem processes [126–128]. For example, divergence in foraging traits of predatory fish species can alter the structure and dynamics of ecological communities by decreasing the mean body size, total biomass, and species richness of crustacean zooplankton [129]. The consequences of phenotypic divergence for ecological processes should be particularly strong when the species under consideration acts as a keystone species or ecosystem engineer [130] or when it is simply very abundant [127]. Amphipods are considered to be key components of limnic food webs, as their shredding activity accelerates leaf fragmentation, produces faecal pellets, and transfers nutrients into secondary production, all of which are vital for maintaining diverse aquatic food webs [131–135]. Hence, rapid evolutionary divergence of amphipods along stream gradients is likely to result in multiple feedbacks between evolutionary and ecological processes [136–138]. For example, *G. roeselii* shows the strongest degree of body size-dependency in leaf litter decomposition rates amongst several species of native and non-native amphipods in the Rhine drainage (see Additional file 1: Figure S2 in ref. [139]). Hence, the strong decline in body size across the stream gradient reported here will have an effect on decomposition rates and food web dynamics. Ecological studies have merely just begun to acknowledge the role of rapid phenotypic divergence in the provisioning of ecosystem services [128, 136, 140], and our present study identifies invasive amphipods as prime candidates for future studies in this direction. Specifically, future studies could quantify decomposition rates [139] of phenotypically divergent populations to explicitly integrate differences in functional responses as a consequence of the observed trait variation and to understand how they translate into differences in ecosystem functioning [140–142].

### Drivers of phenotypic divergence along the examined stream gradients

The native distribution range of *G. roeselii* is characterized by its karst topography, with spatio-temporally diverse ecological conditions along stream gradients [143, 144]. Therefore, *G. roeselii* might have a high potential to inhabit variable environments, either by phenotypic plasticity (see below) or through adaptive evolutionary divergence (local adaptation; see also Additional file 1: Material S1). In support of this idea, a substantial amount of the observed phenotypic trait divergence we found in our dataset could be explained by selection factors that we condensed into principal components. We will base our discussion largely on *a priori* predictions of how single components of the river gradient should affect trait divergence (Table 1), but acknowledge that additional factors that were not assessed here (e.g., predation pressure [145, 146], competition with congeneric species [147]) may have driven parts of the observed divergence.

#### Selection from abiotic factors

Abiotic factors had strong effects on phenotypic divergence of various traits. For instance, we found larger individuals at upstream sites. In arthropods, larger specimens tend to have an increased ability to withstand environmental fluctuation [47, 48]. Temperature regimes are more variable and winter temperatures colder at higher altitudes, and increased tolerance to low temperatures in large-bodied individuals was empirically confirmed for *Gammarus lacustris* [50]. Increased body size at higher altitudes appears to be a widespread pattern in arthropods, unless high seasonality leads to a reduced resource availability and ultimately results in smaller body size at higher altitudes [47, 148]. As a leaf shredding species [139], *G. roeselii* is not resource-limited at upstream sites (i.e., higher altitudes), allowing individuals from those populations to grow to a bigger size.

Counter to our prediction based on the efficiency of oxygen uptake under low oxygen tension [46], we found no response in gill surface area to oxygen conditions; instead we found a pronounced decrease in gill surface areas towards downstream conditions and at sites receiving industrial cooling water. Even though we are lacking quantitative information on pollution loads (other than conductivity as a broad and indirect proxy of total dissolved ions), we argue that increased concentrations of anthropogenic contaminants might explain this pattern ([70]; Table 1). Cooling water often contains additive biocides such as chlorine, corrosion inhibitors, or antifreezers [149]. In support of this interpretation, we found more incidences of intersex at sites that receive cooling water (Additional file 1: Material S4). Biocides are suspected to disturb the hormone balance between androgens and estrogens and facilitate development of both, male and female characteristics at the same time; however, the biochemical pathways that induce intersex are diverse and still remain controversial [106, 150]. Moreover, contaminants of both natural (e.g., from nitrogenous waste products or soil erosion [109]) and anthropogenic origin (e.g., effluents from wastewater treatment plants and agriculture [151–153]) accumulate towards downstream sites. We argue that smaller gill surface areas are adaptive in that they facilitate a reduced uptake of bioaccumulating contaminants (e.g., heavy metals [109]) or to account for changing concentration of ions in the water (i.e., osmoregulatory purposes [154]).

#### Selection from biotic factors

We found systematic co-variation of population demography and densities along the examined river stretches. Specifically, higher population densities were associated with more female biased sex-ratios. The exact mechanisms underlying this patterns could not be examined in the course of our present study. A likely explanation could be sex-distortion through parasites [155], as parasite transmission probably increases with increasing population densities. For example, presence of the microsporidian *Nosema granulosis* is associated with an excess of females in *G. roeselii* broods [156].

Our analyses uncovered divergence of sexually selected male traits towards sites with higher densities and towards female biased sex-ratios. Specifically, the sexual dimorphism in the 1^st^ antennae [71, 112] became more pronounced (Fig. 6c). Male amphipods use their antennae to assess the quality of potential mating partners [72]. They bear high costs of mating due to precopulatory mate guarding [157], which should indeed select for pronounced male mate choosiness [158]. Interestingly, mate choosiness in our study species was stronger in males originating from a high-density site compared to males from a low-density site [113]. Based on the aforementioned considerations, we suggest the following scenario: systematic variation of (unrecognized) ecological factors (e.g., predation pressure [145, 146] and/or competition with congeneric species [147]) along the examined stream sections drove differences in population densities. Mediated by parasitism [156], this altered population demographics, which in turn altered the fitness landscape for sexually selected traits. Moreover, we predicted body size to be positively sexually selected at high conspecific densities [59, 60] (Table 1), but instead found reduced body size in both sexes. This likely reflects elevated resource competition at high population densities translating into reduced somatic growth [159].

Finally, also reproductive traits changed in response to biotic conditions, such as population densities, and we observed a lower fecundity at high conspecific densities and more female biased sex-ratios. A possible adaptive interpretation could be related to the trade-off between offspring size and fecundity [69], where female fecundity is reduced when selection favours bigger offspring [1, 18, 160]. Increased offspring size can be adaptive in highly competitive environments (i.e., at high population densities [68, 161]). Our interpretation remains speculative, however, as the effect of larger offspring size under higher population densities was marginally non-significant (Additional file 1: Figure S2).

#### Selection at invasion fronts?

We further found higher size-corrected fecundity at upstream sites. Our *a priori* predictions for fecundity, based on the existing literature on aquatic invertebrates (Table 1), fail to explain this pattern. Therefore, we argue that selective regimes at upstream sites need to be viewed in light of the species’ colonisation history. Upstream sites in Central Europe were only recently invaded by *G. roeselii* (this study). Individuals on the expanding edge of a population (or invasion front) face a unique selective environment. They encounter native competitors (in this case even congeners [94, 101, 162]) and a new set of abiotic selection factors [163]. Selection arising from this condition may tip the scale of another prominent life-history trade-off, namely the trade-off between investment into reproduction and somatic maintenance, which is related to the trade-off between current and future reproduction [82]. Invaders may thus show more investment into reproduction [164] unless there is a strong trade-off between dispersal abilities and fecundity [165].

### Outlook and future research

Phylogeographic studies on amphipods are becoming common practice to study how past geological events affected macroevolutionary processes [166–168]. By contrast, phenotypic divergence within and between taxonomic groups—as reported here on an intraspecific level for *G. roeselii*—received little attention so far. Uncertainty remains regarding the underlying mechanisms that caused the observed phenotypic variation, and future studies are warranted that address the potential heritability of the traits studied herein and the role of adaptive phenotypic plasticity [87, 169]. Additionally, parts of the observed phenotypic divergence could also arise from non-adaptive plasticity in response to stressful environments [87] (Additional file 1: Material S1). Future studies could provide broad-sense heritability estimates by rearing individuals under common-garden conditions [170], or make an attempt to rear individual broods from known parents to provide narrow-sense heritability estimates (*h*_n_^2^, via mid-parent – offspring regressions [171]). To experimentally address some of the newly generated hypotheses, future studies could use the sister genus *Hyalella*, which is easy to breed under laboratory conditions. *Hyalella* spp. are routinely used in North America as a model organism for ecotoxicological assays [172] and contemporary evolution to anthropogenic sources of selection (pesticide exposure) has been described [173]. On the other hand, the relative role of plastic responses could be addressed experimentally by assessing the variance of traits, for example, in reciprocal transplant experiments [174, 175]. Finally, genome- and transcriptome-screens could provide insights into the underlying mechanisms of phenotypic divergence, and the widespread occurrence of *G. roeselii* offers several independent populations to test for parallel adaptive divergence. Altogether then, our present study identifies amphipods are promising models to study phenotypic diversification along ecological gradients made up by both natural and human-induced selection factors. Furthermore, their key-role in freshwater ecosystems renders amphipods an excellent system in which to investigate feedbacks between evolutionary and ecological processes.

## Additional file

Additional file 1: Material S1. Additional information on sources of phenotypic divergence. Material S2. Additional information on genetic differentiation in *Gammarus roeselii*. Material S3. Additional information on life-history characteristics of *Gammarus roeselii*. Material S4. Additional information on intersexuality. Material S5. Additional information on embryonic developmental stages. Material S6. Information on study sites, additional results and model summaries. **Table S1.** Environmental conditions for each sampling site. **Table S2.** PC scores per season and site. **Table S3.** Numbers of (a) sex-determined specimens for each sampling site with number of intersex individuals and (b) individuals used for measuring phenotypic traits (male ♂, female ♀). **Figure S1.** Relationship of body length to head capsule length. **Figure S2.** Visualization of the marginally non-significant model term in generalized least squares models using offspring-size as the dependent variable. **Table S4.** Body length. **Table S5.** Dry weight. **Table S6.** Gill surface area**. Table S7.** 1^st^ Antennae**. Table S8.** 2^nd^ Antennae. **Table S9.** Gnathopod length. **Table S10.** Fecundity. **Table S11.** Egg size. **Figure S3.** Quantile-quantile (QQ) plots of the model residuals. **Figure S4.** Pictures of sampling sites. **Table S12.** Location of sampling sites. (DOCX 1830 MB)

## Data Availability

The datasets used and analysed for this study are available from the corresponding author upon reasonable request.

## Abbreviations

GLS: Generalized least squares model
PC: Principal component
