# Supplementary material for: Small-scale phenotypic differentiation along complex stream gradients in a non-native amphipod
Source: Front Zool. 2019 Jul 11;16:29. doi: 10.1186/s12983-019-0327-8 (PMC6624920; doi:10.1186/s12983-019-0327-8)
Supplement: Supplementary file 1 — Material S1. Additional information on sources of phenotypic divergence. Material S2. Additional information on genetic differentiation in Gammarus roeselii. Material S3. Additional information on life-history characteristics of Gammarus roeselii. Material S4. Additional information on intersexuality. Material S5. Additional information on embryonic developmental stages. Material S6. Information on study sites, additional results and model summaries. Table S1. Environmental conditions for each sampling site. Table S2. PC scores per season and site. Table S3. Numbers of (a) sex-determined specimens for each sampling site with number of intersex individuals and (b) individuals used for measuring phenotypic traits (male ♂, female ♀). Figure S1. Relationship of body length to head capsule length. Figure S2. Visualization of the marginally non-significant model term in generalized least squares models using offspring-size as the dependent variable. Table S4. Body length. Table S5. Dry weight. Table S6. Gill surface area. Table S7. 1st Antennae. Table S8. 2nd Antennae. Table S9. Gnathopod length. Table S10. Fecundity. Table S11. Egg size. Figure S3. Quantile-quantile (QQ) plots of the model residuals. Figure S4. Pictures of sampling sites. Table S12. Location of sampling sites. (DOCX 1830 MB) [file 12983_2019_327_MOESM1_ESM.docx]

Supplementary Material

**Small-scale phenotypic differentiation along complex stream gradients in a non-native amphipod**

Jonas Jourdan, Kathrin Piro, Alexander Weigand and Martin Plath

**Supplementary Material 1**

**Additional information on sources of phenotypic divergence**

Phenotypic divergence could arise by different processes, namely local adaptation (resulting in evolved differences between populations), adaptive phenotypic plasticity, non-adaptive plasticity, and reversible plasticity (acclimation) [1-3]. Consistency (i.e., predictability) of selection pressure on a certain trait or trait suite is necessary to evolve a locally adapted phenotype [4]. For example, selection at the edge of a species’ geographical range should continuously favour high investment into reproduction (e.g., high fecundity [5]), while strong predation should bring about selection on small body size in prey species [6]. Different sex roles during mate finding, mate acquisition and mate choice should favour sexual dimorphism in traits related to inter-sexual communication [7, 8], and local differences in ecological factors interfering with (or promoting) inter-sexual communication can alter the selective landscape for traits involved in mate finding [7], mate acquisition [8] and mate choice [9].

Adaptive plasticity can occur, for example, when certain physico-chemical parameters fluctuate between seasons and affect physiological processes (e.g., metabolism). Populations may evolve the ability to respond to predictably fluctuating local conditions by altering phenotypic traits [1, 10]. Another driver of phenotypic divergence is non-adaptive plasticity in response to stressful environments. It is characterized by mean phenotypic responses being further away from the favoured optimum than before the stressor was acting [1]. Traits like fat-content or body weight—indicators of body condition—might underlie such non-adaptive plasticity and respond immediately to local stressful conditions [1]. Finally, phenotypic divergence could be the results of reversible plasticity (acclimation). It occurs in both juvenile and mature organisms and involves short (e.g., several days) phenotypic changes that are reversible and may occur repeatably within an individuals’ lifetime [3].

**Supplementary Material 2**

**Additional information on genetic differentiation in *Gammarus roeselii***

The distribution of the family Gammaridae in general (and of the genus *Gammarus* in particular) is broad, centred in Europe, but extending into China and North America [11]. Amphipods of the genus *Gammarus* are a characteristic component of aquatic communities in surface waters of northern Eurasia and America [107, 108]. They are often abundant and constitute an important functional component of the benthic decomposer community [42, 43, 109, 110]. Within their native range, gammarids often show strong genetic differentiation, with restricted gene flow between populations, even at spatial scales of only few kilometres [12, 13]. Several amphipods in Central Europe [12, 14-16] and especially in adjacent regions, such as the Balkans and the Carpathian Mountains [14, 17, 18], have been shown to comprise cryptic genetic diversity, with numerous ancient phylogenetic lineages reflecting past geological events [19-21].

Although *G. roeselii* is known to comprise multiple cryptic lineages, *G. roeselii* are less genetically diverse in their distribution range outside the Balkan area [17, 22] compared to native congeners (such as *G. fossarum* [12, 14, 15]), and a single widespread evolutionary lineage has been reported for Central Europe [17]. This allowed us to study intraspecific phenotypic differentiation while overcoming potentially confounding effects of phylogenetic distance/relatedness among cryptic lineages. Small-scale population genetic studies on *G. roeselii* are as yet missing; however, the widespread occurrence of a single evolutionary lineage in Central Europe [17] suggest a higher dispersion potential compared to native congeners.

**Supplementary Material 3**

**Additional information on life-history characteristics of *Gammarus roeselii***

At 20°C, *G. roeselii* attains sexual maturity after 85 days*,* after nine to ten moults and at a body length of 8 – 9 mm [23]. The smallest reproductively active female in our dataset was even smaller, with a body size of 7.85 mm. Under laboratory conditions and a constant temperature of 15°C, the life span of *G. roeselii* exceeds 600 days [23]. Adult females presumably moult approximately six to ten times, usually releasing a brood at each moult. They reproduce throughout most of the year, but breeding is reduced during the winter months [24]. Growth of *G. roeselii* from birth to senescence or death is described by a logistic function, as individuals continue to grow after maturation until they reach the inflection point of the growth curve [23, 25].

Amphipods have a direct development (i.e., no free-swimming larval stage [26]). Females carry their developing broods in an external brood pouch—the marsupium—formed by four pairs of oostegites (also called ‘brood plates’; Figure 2g in the main text), which can be differentiated from the gills by their setae [26, 27] (Figure 2h in the main text). This behaviour functionally renders amphipods ‘live-bearers’ and allows comparative analyses of offspring-related life history traits on an individual level similar to studies on mammals [28] and live-bearing fishes [29, 30].

**Supplementary Material 4**

**Additional information on intersexuality**

***Additional background information***

While amphipods are usually gonochoristic, we occasionally found individuals with both, male and female sexual characteristics [31]. Specifically, intersexual individuals possessed both genital papillae and oostegites [32]. Intersexuality is known from at least 20 different amphipod species, but has not yet been described for *G. roeselii* [31]. The triggers of intersexuality in amphipods are not yet fully understood and include environmental contamination by estrogenic and organotin endocrine-disrupting chemicals (EDCs), infection by microsporidian parasites, and abnormalities in genetic/environmental sex determination [31, 32].

***Additional results***

As reported in other studies [31], intersexuality was a rare event across sampling sites that could be linked to anthropogenic disturbance. To compare the frequency of intersexual individuals between thermally polluted and non-polluted sites, we used a chi-square test. Overall, we collected 3,241 individuals (1,162 at sites receiving artificially heated cooling water and 2,079 at thermally unimpacted sites), 16 of which showed an intersex phenotype (Figure 2b in the main text). Notably, 12 of the intersexual individuals were caught at sites receiving cooling water (1.03% of individuals), while thermally unimpacted sites had an intersex rate of only 0.19%. A chi-square test confirmed a significant difference between thermally polluted and unpolluted sites (χ^2^ = 10.58, *p* = 0.001). The probability (odds ratio) of intersex was 5.37 (95% CI: 1.73, 16.68) times higher at thermally polluted than non-polluted sites.

**Supplementary Material 5**

**Additional information on embryonic developmental stages**

Identifying developmental stages in our present study was necessary to account for ontogenetic changes of egg size/volume. We distinguished the following seven developmental stages: newly laid eggs are surrounded by a gelatinous mucus layer, which is secreted by the female during oviposition and dissolves after several hours. During this time fertilization takes place [33]. After fertilization each batch of eggs is surrounded by a membranous sac. Newly fertilized eggs (stage 1) are oval and have two membranes, the chorion and the second embryonic layer, which is tightly connected to the yolk. At this stage, no cell cleavage is observed [34] (Figure 3b in the main text). At stage 2 the two membranes still exist, but the cells divide (holoblastic cleavage) and form large, non-nucleated and pigmented yolk cells [34] (Figure 3c). Stage 3 is characterized by the appearance of a caudal groove (or ventral cleft [35]), which divides the body of the developing embryo into cephalothorax and abdomen (Figure 3d). During stage 4, body appendages develop, and the embryo forms a ‘comma-like’ body shape. The dorsal organ (formed by a number of elongated endodermal cells) has now reached its maximum size [25, 34]. We could not observe any body pigmentation [34], likely because it was lost in our alcohol-fixed samples (Figure 3e). Stage 5 is characterized by the emergence of the first eye pigments, fully developed, segmented body appendages, and the regression of the dorsal organ (Figure 3f). At stage 6 the embryo’s body is fully formed. Compound eyes are completely developed and clearly visible, even after fixation in ethanol (Figure 3g). Stage 7 describes hatched juveniles, which can still be present in the marsupium for a short period of time [26, 34] (Figure 3h). Stage 7 differs from stage 6 by the ruptured chorion, which hatchlings leave or even consume [34].

**Table S1:** Environmental conditions for each sampling site

|  | Site ID | River | Water temperature [°C] | Oxygen content [mg l^-1^] | Conductivity [μS cm^-1^] | Altitude [m] | Sex ratio | Width | Depth | Flow velocity | Population density | Received industrial cooling water |
| --- | --- | --- | --- | --- | --- | --- | --- | --- | --- | --- | --- | --- |
| Winter | 1 | Erft | 11.4 | 9.32 | 889 | 41 | 3.4 | 3 | 2 | 2 | 2 | 1 |
|  | 2 | Erft | 12 | 9.82 | 868 | 48 | 3 | 2 | 2 | 2 | 3 | 1 |
|  | 3 | Erft | 13.7 | 9.37 | 870 | 56 | 3.1 | 3 | 2 | 2 | 3 | 1 |
|  | 4 | Erft | 4.5 | 13.26 | 1025 | 82 | 2.1 | 2 | 1 | 2 | 1 | 0 |
|  | 5 | Erft | 4 | 14 | 743 | 188 | 3.8 | 1 | 1 | 2 | 2 | 0 |
|  | 6 | Kinzig | 6.1 | 10.4 | 412 | 114 | 1.6 | 2 | 2 | 1 | 2 | 0 |
|  | 7 | Kinzig | 6.6 | 10.14 | 363 | 134 | 2.5 | 2 | 2 | 2 | 1 | 0 |
|  | 8 | Kinzig | 7.8 | 9.38 | 277 | 178 | 1.2 | 3 | 3 | 1 | 2 | 0 |
|  | 9 | Kinzig | 6.9 | 9.81 | 379 | 224 | 3.3 | 1 | 1 | 2 | 2 | 0 |
|  | 10 | Gründau | 7.1 | 11.08 | 271 | 135 | 2.1 | 1 | 1 | 3 | 3 | 0 |
|  | 11 | Gründau | 6.1 | 11.9 | 189 | 163 | 1.8 | 1 | 1 | 2 | 2 | 0 |
|  | 12 | Bracht | 6.7 | 9.98 | 185 | 149 | 2.3 | 1 | 2 | 2 | 1 | 0 |
|  | 13 | Bracht | 6.1 | 11.5 | 146 | 321 | 3.3 | 1 | 1 | 2 | 3 | 0 |
|  | 14 | Salz | 5.4 | 10.1 | 131 | 378 | 1.2 | 1 | 2 | 2 | 2 | 0 |
|  | 15 | Ulmbach | 5.6 | 9.5 | 214 | 402 | 1.6 | 1 | 1 | 2 | 1 | 0 |
|  | 16 | Schwarzbach | 6.8 | 9.5 | 214 | 228 | 1.3 | 1 | 2 | 2 | 2 | 0 |
| Summer | 1 | Erft | 23.4 | 8.21 | 830 | 41 | 1.4 | 2 | 3 | 2 | 2 | 1 |
|  | 2 | Erft | 24 | 8.18 | 858 | 48 | 1.8 | 2 | 3 | 2 | 3 | 1 |
|  | 3 | Erft | 23.7 | 8.6 | 840 | 56 | 2 | 3 | 3 | 2 | 3 | 1 |
|  | 4 | Erft | 22.9 | 9.01 | 848 | 82 | 0.4 | 2 | 2 | 2 | 1 | 0 |
|  | 5 | Erft | 21.7 | 8.56 | 695 | 188 | 4.2 | 1 | 1 | 2 | 2 | 0 |
|  | 6 | Kinzig | 19.5 | 6.95 | 582 | 114 | 2.1 | 2 | 2 | 2 | 2 | 0 |
|  | 7 | Kinzig | 19.6 | 7.66 | 540 | 134 | 2.4 | 3 | 2 | 2 | 2 | 0 |
|  | 8 | Kinzig | - | - | - | - | - | - | - | - | - | - |
|  | 9 | Kinzig | 16 | 9.38 | 507 | 224 | 5.5 | 1 | 1 | 2 | 2 | 0 |
|  | 10 | Gründau | 18.5 | 7.56 | 304 | 135 | 1.6 | 1 | 1 | 2 | 2 | 0 |
|  | 11 | Gründau | 17.8 | 8.6 | 165 | 163 | 0.6 | 1 | 1 | 2 | 1 | 0 |
|  | 12 | Bracht | 14.3 | 7.61 | 238 | 149 | 1.5 | 1 | 1 | 3 | 1 | 0 |
|  | 13 | Bracht | 15.2 | 9.43 | 175 | 321 | 3.8 | 1 | 1 | 2 | 2 | 0 |
|  | 14 | Salz | 13.8 | 9.45 | 195 | 378 | 2 | 1 | 1 | 1 | 3 | 0 |
|  | 15 | Ulmbach | 14.4 | 8.85 | 249 | 402 | 1.3 | 1 | 1 | 1 | 3 | 0 |
|  | 16 | Schwarzbach | 16.6 | 8.85 | 420 | 228 | 1.2 | 1 | 1 | 1 | 2 | 0 |

**Table S2**: PC scores per season and site

|  | Site | Environmental PC1 | Environmental PC2 | Environmental PC3 | Environmental PC4 |
| --- | --- | --- | --- | --- | --- |
| Winter | 1 | 1.69 | 0.72 | -0.63 | -0.86 |
|  | 2 | 1.32 | 1.29 | -0.26 | -0.61 |
|  | 3 | 1.66 | 1.15 | -0.12 | -0.63 |
|  | 4 | 0.61 | -0.12 | -1.98 | -0.7 |
|  | 5 | -0.46 | 1.17 | 0.57 | -0.99 |
|  | 6 | 0.26 | -1.15 | 0.85 | -0.96 |
|  | 7 | 0.29 | -0.57 | -0.93 | -1.07 |
|  | 8 | 0.86 | -2.48 | -0.55 | -0.99 |
|  | 9 | -0.62 | 0.81 | -0.57 | -0.53 |
|  | 10 | -0.42 | 1.66 | -0.13 | -0.73 |
|  | 11 | -0.7 | 0.12 | 0.96 | -1.07 |
|  | 12 | -0.22 | -0.55 | -0.72 | -1 |
|  | 13 | -1.14 | 1.22 | 1.42 | -0.7 |
|  | 14 | -0.84 | -0.59 | 1.46 | -1.05 |
|  | 15 | -1.19 | -0.64 | 0.8 | -1.08 |
|  | 16 | -0.46 | -0.44 | 1.28 | -1.16 |
| Summer | 1 | 1.06 | -0.7 | -0.34 | 0.56 |
|  | 2 | 1.53 | 0.31 | 0.07 | 0.67 |
|  | 3 | 2.07 | 0.29 | 3.34 | 2.14 |
|  | 4 | 1.22 | -1.11 | -0.72 | 0.6 |
|  | 5 | -0.5 | 1.19 | -0.79 | 1.09 |
|  | 6 | 0.33 | -0.22 | -0.22 | 0.63 |
|  | 7 | 0.6 | -0.3 | -0.36 | 0.57 |
|  | 9 | -0.79 | 1.64 | -1.1 | 0.98 |
|  | 10 | -0.57 | 0.52 | 0.22 | 1.05 |
|  | 11 | -0.8 | -1.11 | 0.59 | 0.28 |
|  | 12 | -0.65 | 0.1 | -0.53 | 0.24 |
|  | 13 | -1.14 | 0.74 | -0.52 | 1.07 |
|  | 14 | -1.17 | -0.83 | -0.4 | 1.44 |
|  | 15 | -1.16 | -1.11 | -0.23 | 1.44 |
|  | 16 | -0.66 | -1 | -0.47 | 1.34 |

Table S3: Numbers of (a) sex-determined specimens for each sampling site with number of intersex individuals and (b) individuals used for measuring phenotypic traits (male ♂, female ♀). The number of embryo carrying females is given in brackets.

|  | **Site ID** | **(a) Sex determined** | | **(b) Traits measured** | | |
| --- | --- | --- | --- | --- | --- | --- |
|  |  | **Overall sexed specimens** | **Number of intersex** | **Total** | ♂ | ♀ |
| **Winter** | 1 | 72 | 1 | 71 | 16 | 55 (22) |
|  | 2 | 162 | 1 | 70 | 21 | 49 (21) |
|  | 3 | 275 | 2 | 65 | 21 | 44 (26) |
|  | 4 | 52 | 0 | 52 | 17 | 35 (6) |
|  | 5 | 63 | 0 | 63 | 13 | 50 (5) |
|  | 6 | 86 | 0 | 50 | 25 | 25 (1) |
|  | 7 | 39 | 0 | 39 | 11 | 28 (1) |
|  | 8 | 75 | 0 | 50 | 28 | 22 (1) |
|  | 9 | 81 | 0 | 64 | 16 | 48 (2) |
|  | 10 | 110 | 1 | 52 | 20 | 32 (2) |
|  | 11 | 90 | 0 | 50 | 22 | 28 (0) |
|  | 12 | 53 | 0 | 53 | 16 | 37 (0) |
|  | 13 | 137 | 1 | 69 | 21 | 48 (25) |
|  | 14 | 69 | 0 | 53 | 27 | 26 (1) |
|  | 15 | 71 | 0 | 50 | 21 | 29 (0) |
|  | 16 | 54 | 0 | 53 | 23 | 30 (1) |
| **Spring** | 1 | 103 | 3 | 57 | 25 | 32 (29) |
|  | 2 | 213 | 5 | 54 | 22 | 32 (25) |
|  | 3 | 337 | 0 | 56 | 23 | 33 (29) |
|  | 4 | 28 | 0 | 28 | 20 | 8 (5) |
|  | 5 | 89 | 0 | 53 | 16 | 37 (35) |
|  | 6 | 88 | 0 | 50 | 21 | 29 (28) |
|  | 7 | 92 | 0 | 50 | 22 | 28 (26) |
|  | 8 | 2 | 0 | 0 | - | - |
|  | 9 | 123 | 0 | 48 | 19 | 29 (29) |
|  | 10 | 113 | 1 | 51 | 24 | 27 (27) |
|  | 11 | 72 | 0 | 50 | 26 | 24 (6) |
|  | 12 | 58 | 0 | 50 | 22 | 28 (24) |
|  | 13 | 86 | 0 | 45 | 17 | 28 (26) |
|  | 14 | 144 | 0 | 50 | 22 | 28 (26) |
|  | 15 | 114 | 0 | 52 | 23 | 29 (28) |
|  | 16 | 90 | 1 | 50 | 24 | 26 (26) |


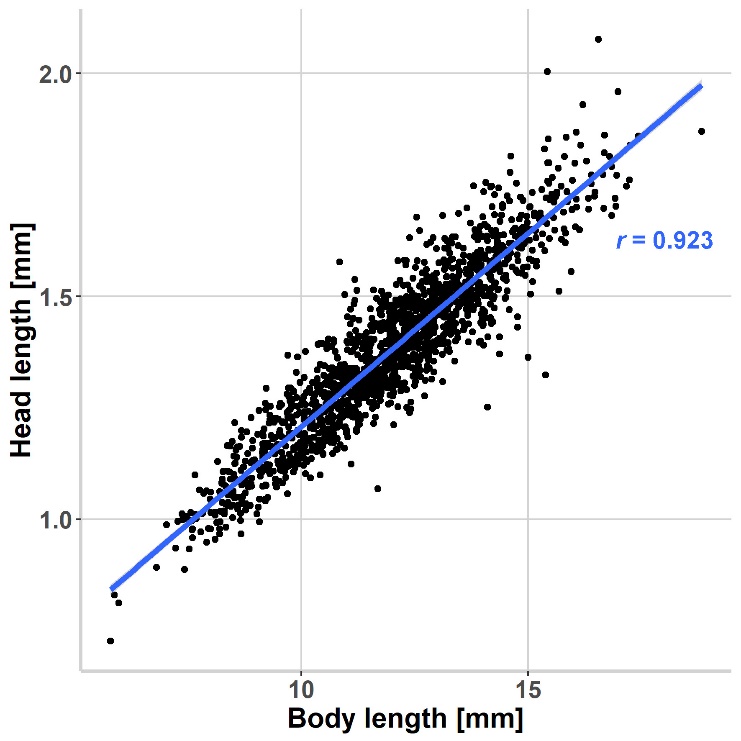


**Figure S1: Relationship of body length to head capsule length.** Both traits strongly correlate with each other (Pearson *r* = 0.923).


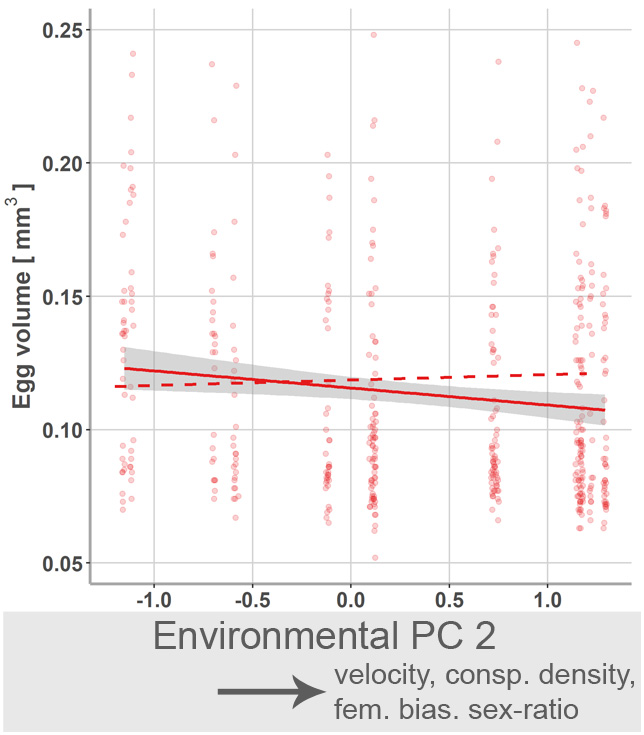


**Figure S2: Visualization of the marginally non-significant model term in generalized least squares models using offspring-size as the dependent variable**. Solid lines show linear relationship of raw data, while dashed lines represent the linear relationships based on predicted values that were adjusted for other predictors in the model (e.g., female body length and egg developmental stage).

**Model summaries**

**Table S4: Body length**

(Correlation structure AR(1), formula: ~1. Parameter estimate(s): Phi 0.284)

|  | Value | SE | *t*-value | *p*-value |
| --- | --- | --- | --- | --- |
| (Intercept) | 11.6840 | 0.10 | 121.02 | < 0.001 |
| Environmental PC 1 | -0.4707 | 0.10 | -4.89 | < 0.001 |
| Environmental PC 2 | -0.1765 | 0.09 | -2.00 | 0.046 |
| Environmental PC 3 | -0.1621 | 0.10 | -1.57 | 0.116 |
| Environmental PC 4 | -0.0990 | 0.12 | -0.85 | 0.398 |
| Sexmale | 0.7528 | 0.12 | 6.32 | < 0.001 |
| Sex male × env. PC 1 | 0.0034 | 0.12 | 0.03 | 0.978 |
| Sex male × env. PC 2 | -0.1138 | 0.11 | -1.04 | 0.298 |
| Sex male × env. PC 3 | 0.1861 | 0.14 | 1.37 | 0.171 |
| Sex male × env. PC 4 | 0.0445 | 0.15 | 0.30 | 0.766 |

**Table S5: Dry weight**

(Correlation structure AR(1), formula: ~1. Parameter estimate(s): Phi 0.186)

|  | Value | SE | *t*-value | *p*-value |
| --- | --- | --- | --- | --- |
| (Intercept) | -9.2288 | 0.25 | -37.31 | < 0.001 |
| Environmental PC 1 | -0.0907 | 0.07 | -1.24 | 0.217 |
| Environmental PC 2 | -0.2109 | 0.07 | -3.15 | 0.002 |
| Environmental PC 3 | 0.0088 | 0.08 | 0.11 | 0.910 |
| Environmental PC 4 | 0.2230 | 0.09 | 2.52 | 0.012 |
| Sex male | 0.0350 | 0.10 | 0.35 | 0.728 |
| Body length | 1.3615 | 0.02 | 67.26 | < 0.001 |
| Sex male × env. PC 1 | -0.1484 | 0.10 | -1.45 | 0.148 |
| Sex male × env. PC 2 | 0.1222 | 0.09 | 1.33 | 0.184 |
| Sex male × env. PC 3 | -0.1136 | 0.11 | -0.99 | 0.321 |
| Sex male × env. PC 4 | -0.1642 | 0.13 | -1.31 | 0.190 |

**Table S6: Gill surface area**

(Correlation structure AR(1), formula: ~1. Parameter estimate(s): Phi 0.225)

|  | Value | SE | *t*-value | *p*-value |
| --- | --- | --- | --- | --- |
| (Intercept) | -6.1830 | 0.32 | -19.16 | < 0.001 |
| Environmental PC 1 | -0.3524 | 0.09 | -3.90 | < 0.001 |
| Environmental PC 2 | -0.0721 | 0.08 | -0.92 | 0.359 |
| Environmental PC 3 | 0.0014 | 0.11 | 0.01 | 0.990 |
| Environmental PC 4 | 0.3528 | 0.11 | 3.22 | 0.001 |
| Body length | 1.1341 | 0.03 | 44.87 | < 0.001 |

**Table S7: 1^st^ Antennae**

(Correlation structure AR(1), formula: ~1. Parameter estimate(s): Phi 0.192)

|  | Value | SE | *t*-value | *p*-value |
| --- | --- | --- | --- | --- |
| (Intercept) | -0.0514 | 0.09 | -0.60 | 0.547 |
| Environmental PC 1 | 0.2003 | 0.03 | 7.94 | < 0.001 |
| Environmental PC 2 | 0.0138 | 0.02 | 0.59 | 0.553 |
| Environmental PC 3 | 0.1575 | 0.03 | 5.85 | < 0.001 |
| Environmental PC 4 | 0.1915 | 0.03 | 6.32 | < 0.001 |
| Sex male | 0.4230 | 0.03 | 12.36 | < 0.001 |
| Body length | 0.4640 | 0.01 | 66.25 | < 0.001 |
| Sex male × env. PC 1 | -0.0100 | 0.03 | -0.29 | 0.775 |
| Sex male × env. PC 2 | 0.0754 | 0.03 | 2.38 | 0.017 |
| Sex male × env. PC 3 | -0.0123 | 0.04 | -0.31 | 0.755 |
| Sex male × env. PC 4 | -0.0317 | 0.04 | -0.74 | 0.456 |

**Table S8: 2^nd^ Antennae**

(Correlation structure AR(1), formula: ~1. Parameter estimate(s): Phi 0.196)

|  | Value | SE | *t*-value | *p*-value |
| --- | --- | --- | --- | --- |
| (Intercept) | -1.0798 | 0.06 | -19.15 | < 0.001 |
| Environmental PC 1 | 0.1180 | 0.02 | 7.00 | < 0.001 |
| Environmental PC 2 | 0.0262 | 0.02 | 1.70 | 0.089 |
| Environmental PC 3 | 0.0607 | 0.02 | 3.38 | 0.001 |
| Environmental PC 4 | 0.0752 | 0.02 | 3.69 | < 0.001 |
| Sex male | 0.8508 | 0.02 | 37.17 | < 0.001 |
| Body length | 0.3234 | 0.00 | 70.09 | < 0.001 |
| Sex male × env. PC 1 | -0.0602 | 0.02 | -2.58 | 0.010 |
| Sex male × env. PC 2 | -0.0046 | 0.02 | -0.22 | 0.828 |
| Sex male × env. PC 3 | 0.0014 | 0.03 | 0.05 | 0.959 |
| Sex male × env. PC 4 | -0.0208 | 0.03 | -0.73 | 0.464 |

**Table S9: Gnathopod length**

(Correlation structure AR(1), formula: ~1. Parameter estimate(s): Phi 0.202)

|  | Value | SE | *t*-value | *p*-value |
| --- | --- | --- | --- | --- |
| (Intercept) | -0.0558 | 0.04 | -1.46 | 0.145 |
| Environmental PC 1 | -0.0012 | 0.01 | -0.12 | 0.906 |
| Environmental PC 2 | 0.0076 | 0.01 | 0.84 | 0.400 |
| Environmental PC 3 | 0.0067 | 0.01 | 0.57 | 0.569 |
| Environmental PC 4 | -0.0003 | 0.01 | -0.02 | 0.983 |
| Body length | 0.1676 | 0.00 | 56.48 | < 0.001 |

**Table S10: Fecundity**

(Correlation structure AR(1), formula: ~1. Parameter estimate(s): Phi 0.095)

|  | Value | SE | *t*-value | *p*-value |
| --- | --- | --- | --- | --- |
| (Intercept) | -27.2202 | 4.45 | -6.12 | < 0.001 |
| Environmental PC 1 | -3.5901 | 0.80 | -4.48 | < 0.001 |
| Environmental PC 2 | -6.9782 | 0.79 | -8.84 | < 0.001 |
| Environmental PC 3 | -1.5315 | 0.89 | -1.72 | 0.087 |
| Environmental PC 4 | -1.2282 | 1.03 | -1.20 | 0.233 |
| Body length | 5.2272 | 0.36 | 14.41 | < 0.001 |

**Table S11: Egg size**

(No autocorrelation present)

|  | Value | SE | *t*-value | *p*-value |
| --- | --- | --- | --- | --- |
| (Intercept) | 0.0066 | 0.01 | 0.87 | 0.386 |
| Environmental PC 1 | -0.0014 | 0.00 | -1.21 | 0.226 |
| Environmental PC 2 | 0.0020 | 0.00 | 1.75 | 0.082 |
| Environmental PC 3 | 0.0000 | 0.00 | -0.03 | 0.978 |
| Environmental PC 4 | -0.0002 | 0.00 | -0.13 | 0.894 |
| Main egg stage | 0.0276 | 0.00 | 42.25 | < 0.001 |
| Body length | 0.0021 | 0.00 | 3.68 | < 0.001 |


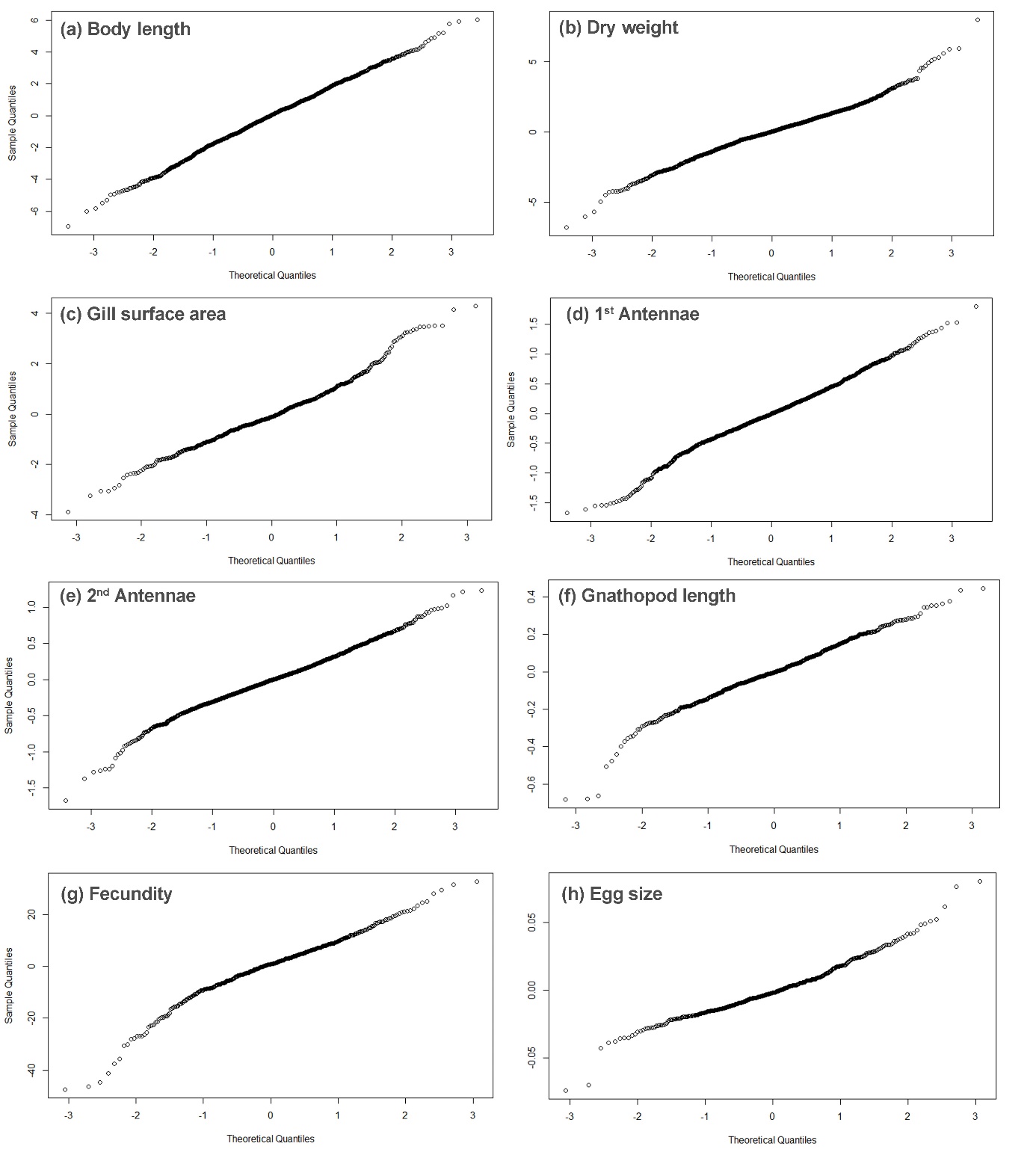


**Figure S3: Quantile‐quantile (QQ) plots of the model residuals**.


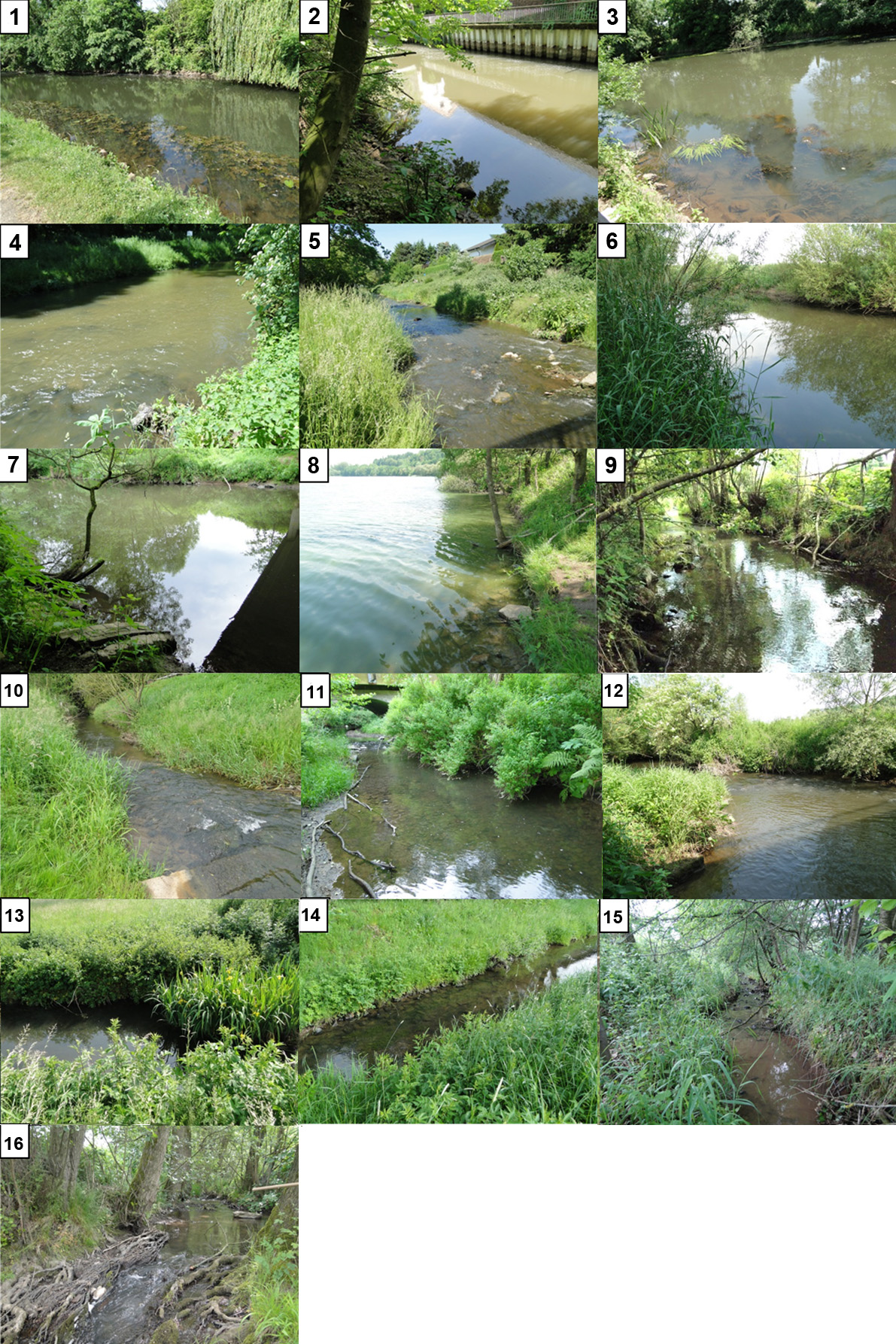


Figure S4: Sampling sites.

**Table S12**: Location of sampling sites

| Site ID | River | Latitude | Longitude |
| --- | --- | --- | --- |
| 1 | Erft | 51.159953 | 6.688182 |
| 2 | Erft | 51.131848 | 6.635366 |
| 3 | Erft | 51.044860 | 6.568722 |
| 4 | Erft | 50.896942 | 6.707853 |
| 5 | Erft | 50.629338 | 6.8055 |
| 6 | Kinzig | 50.152614 | 9.011502 |
| 7 | Kinzig | 50.208652 | 9.228479 |
| 8 | Kinzig | 50.304161 | 9.424094 |
| 9 | Kinzig | 50.346335 | 9.552046 |
| 10 | Gründau | 50.212972 | 9.101777 |
| 11 | Gründau | 50.248833 | 9.155611 |
| 12 | Bracht | 50.278013 | 9.309581 |
| 13 | Bracht | 50.377010 | 9.270373 |
| 14 | Salz | 50.416649 | 9.363432 |
| 15 | Ulmbach | 50.390583 | 9.400805 |
| 16 | Schwarzbach | 50.358823 | 9.551937 |

**Supplementary References**

1. Ghalambor CK, McKay JK, Carroll SP, Reznick DN: **Adaptive versus non-adaptive phenotypic plasticity and the potential for contemporary adaptation in new environments**. *Funct Ecol* 2007, **21**(3):394-407.

2. Gilbert SF, Epel D: **Ecological Developmental Biology: The Environmental Regulation of Development, Health, and Evolution**, second edition edn. Sunderland, MA, USA.: Sinauer Associates Inc, USA.; 2015.

3. Beaman JE, White CR, Seebacher F: **Evolution of plasticity: mechanistic link between development and reversible acclimation**. *Trends Ecol Evol* 2016, **31**(3):237-249.

4. Kawecki TJ, Ebert D: **Conceptual issues in local adaptation**. *Ecol Lett* 2004, **7**(12):1225-1241.

5. Riesch R, Martin RA, Diamond SE, Jourdan J, Plath M, Brian Langerhans R: **Thermal regime drives a latitudinal gradient in morphology and life history in a livebearing fish**. *Biol J Linn Soc* 2018, **125**(1):126-141.

6. Blanckenhorn WU: **The evolution of body size: what keeps organisms small?** *The quarterly review of biology* 2000, **75**(4):385-407.

7. Parker G, Partridge L: **Sexual conflict and speciation**. *Philosophical Transactions of the Royal Society of London Series B: Biological Sciences* 1998, **353**(1366):261-274.

8. Panhuis TM, Butlin R, Zuk M, Tregenza T: **Sexual selection and speciation**. *Trends Ecol Evol* 2001, **16**(7):364-371.

9. Lipkowski K, Plath M, Klaus S, Sommer-Trembo C: **Population density affects morphology and male mate choosiness in the mate-guarding amphipod *Gammarus roeselii* (Crustacea: Amphipoda)**. in press.

10. Agrawal AA: **Phenotypic plasticity in the interactions and evolution of species**. *Science* 2001, **294**(5541):321-326.

11. Väinölä R, Witt J, Grabowski M, Bradbury JH, Jazdzewski K, Sket B: **Global diversity of amphipods (Amphipoda; Crustacea) in freshwater**. *Hydrobiologia* 2008, **595**(1):241-255.

12. Weiss M, Leese F: **Widely distributed and regionally isolated! Drivers of genetic structure in *Gammarus fossarum* in a human-impacted landscape**. *BMC Evol Biol* 2016, **16**(1):153.

13. Alp M, Keller I, Westram A, Robinson CT: **How river structure and biological traits influence gene flow: a population genetic study of two stream invertebrates with differing dispersal abilities**. *Freshw Biol* 2012, **57**(5):969-981.

14. Weiss M, Macher JN, Seefeldt MA, Leese F: **Molecular evidence for further overlooked species within the *Gammarus fossarum* complex (Crustacea: Amphipoda)**. *Hydrobiologia* 2014, **721**(1):165-184.

15. Westram AM, Jokela J, Baumgartner C, Keller I: **Spatial distribution of cryptic species diversity in European freshwater amphipods (*Gammarus fossarum*) as revealed by pyrosequencing**. *Plos One* 2011, **6**(8):e23879.

16. Lagrue C, Wattier R, Galipaud M, Gauthey Z, Rullmann JP, Dubreuil C, Rigaud T, Bollache L: **Confrontation of cryptic diversity and mate discrimination within *Gammarus pulex* and *Gammarus fossarum* species complexes**. *Freshw Biol* 2014, **59**(12):2555-2570.

17. Grabowski M, Mamos T, Bącela-Spychalska K, Rewicz T, Wattier RA: **Neogene paleogeography provides context for understanding the origin and spatial distribution of cryptic diversity in a widespread Balkan freshwater amphipod**. *PeerJ* 2017, **5**:e3016.

18. Copilaş-Ciocianu D, Petrusek A: **The southwestern Carpathians as an ancient centre of diversity of freshwater gammarid amphipods: insights from the *Gammarus fossarum* species complex**. *Mol Ecol* 2015, **24**(15):3980-3992.

19. Cristescu ME, Hebert PD, Onciu TM: **Phylogeography of Ponto‐Caspian crustaceans: a benthic–planktonic comparison**. *Mol Ecol* 2003, **12**(4):985-996.

20. Grigorovich IA, Therriault TW, MacIsaac HJ: **History of aquatic invertebrate invasions in the Caspian Sea**. In: *Marine bioinvasions: Patterns, processes and perspectives.* Springer; 2003: 103-115.

21. Mamos T, Wattier R, Burzyński A, Grabowski M: **The legacy of a vanished sea: a high level of diversification within a European freshwater amphipod species complex driven by 15 My of Paratethys regression**. *Mol Ecol* 2016, **25**(3):795-810.

22. Cormier A, Wattier R, Teixeira M, Rigaud T, Cordaux R: **The complete mitochondrial genome of *Gammarus roeselii* (Crustacea, Amphipoda): insights into mitogenome plasticity and evolution**. *Hydrobiologia* 2018, **825**(1):197-210.

23. Pöckl M: **Effects of temperature, age and body size on moulting and growth in the freshwater amphipods *Gammarus fossarum* and *G. roeseli***. *Freshw Biol* 1992, **27**(2):211-225.

24. Pöckl M, Webb BW, Sutcliffe DW: **Life history and reproductive capacity of *Gammarus fossarum* and *G. roeseli* (Crustacea: Amphipoda) under naturally fluctuating water temperatures: a simulation study**. *Freshw Biol* 2003, **48**(1):53-66.

25. Pöckl M: **Reproductive potential and lifetime potential fecundity of the freshwater amphipods *Gammarus fossarum* and *G. roeseli* in Austrian streams and rivers**. *Freshw Biol* 1993, **30**(1):73-91.

26. Sutcliffe DW: **Reproduction in *Gammarus* (Crustacea, Amphipoda): basic processes**. In: *Freshwater Forum: 2010*.

27. Sheader M: **Factors influencing egg size in the gammarid amphipod *Gammarus insensibilis***. *Marine Biology* 1996, **124**(4):519-526.

28. Gaillard J-M, Festa-Bianchet M, Yoccoz N, Loison A, Toigo C: **Temporal variation in fitness components and population dynamics of large herbivores**. *Annu Rev Ecol Syst* 2000, **31**(1):367-393.

29. Reznick D, Endler JA: **The impact of predation on life history evolution in Trinidadian guppies (*Poecilia reticulata*)**. *Evolution* 1982, **36**(1):160-177.

30. Goodwin NB, Dulvy NK, Reynolds JD: **Life-history correlates of the evolution of live bearing in fishes**. *Philosophical Transactions of the Royal Society B: Biological Sciences* 2002, **357**(1419):259.

31. Grilo TF, Rosa R: **Intersexuality in aquatic invertebrates: prevalence and causes**. *Sci Total Environ* 2017, **592**:714-728.

32. Ford AT: **Intersexuality in Crustacea: an environmental issue?** *Aquatic Toxicology* 2012, **108**:125-129.

33. Sexton E: **On the rearing and breeding of *Gammarus* in laboratory conditions**. *J Mar Biol Assoc U K* 1928, **15**(1):33-55.

34. McCahon C, Pascoe D: **Increased sensitivity to cadmium of the freshwater amphipod *Gammarus pulex* (L.) during the reproductive period**. *Aquatic Toxicology* 1988, **13**(3):183-193.

35. Steele DH, Steele VJ: **The biology of *Gammarus* (Crustacea, Amphipoda) in the northwestern Atlantic. I. *Gammarus duebeni* Lillj**. *Can J Zool* 1969, **47**(2):235-244.
